# Intracrine androgen biosynthesis, metabolism and action revisited

**DOI:** 10.1016/j.mce.2017.08.016

**Published:** 2018-04-15

**Authors:** Lina Schiffer, Wiebke Arlt, Karl-Heinz Storbeck

**Affiliations:** a Institute of Metabolism and Systems Research, University of Birmingham, Edgbaston, Birmingham B15 2TT, UK; b Department of Biochemistry, Stellenbosch University, Stellenbosch 7600, South Africa

**Keywords:** Androgens, Intracrinology, Steroid biosynthesis, Hormone-dependent cancer, Testosterone, 11-oxygenated androgens, 11KA4, 11-keto-androstenedione, 11KDHT, 11-keto-5α-dihydrotestosterone, 11KT, 11-keto-testosterone, 11OHA4, 11β-hydroxy-androstenedione, 11OHDHT, 11β-hydroxy-5α-dihydrotestosterone, 11OHT, 11β-hydroxy-testosterone, 17αHP, 5α-pregnane-17α-ol-3,20-one, 17OHPROG, 17α-hydroxy-progesterone, 3α-adiol, 5α-androstan-3α,17β-diol, 5α-dione, 5α-androstane-3,17-dione, 5-diol, androst-5-ene-3β,17β-diol, A4, androstenedione (androst-4-ene-3,17-dione), AKR, aldo-keto reductase, AR, androgen receptor, AST, androsterone (5α-androstan-3α-ol-17-one), CHOL, cholesterol, CRPC, castration resistant prostate cancer, CYP, cytochrome P450, DHEA, dehydroepiandrosterone (androst-5-ene-3β-ol-17-one), DHEAS, dehydroepiandrosterone sulfate, EpiAST, 5α-androstan-3β-ol-17-one, EpiT, epitestosterone (17α-hydroxy-testosterone, androst-4-ene-17α-ol-3-one), E1, estrone, E1S, estrone sulfate, E2, estradiol, ETIO, etiochonanolone (5β-androstan-3α-ol-17-one), DHT, 5α-dihydrotestosterone (5α-androstan-17β-ol-3-one), HSD, hydroxysteroid dehydrogenase, OATP, organic anion-transporting polypeptide, PAPS, 3′-phospho-adenosine-5′-phosphosulfate, PCOS, polycystic ovary syndrome, Pdiol, 5α-pregnane-3α,17α-diol-20-one, PORD, cytochrome P450 oxidoreductase deficiency, PREG, pregnenolone, PROG, progesterone, StAR, steroidogenic acute regulatory protein, SHBG, sex hormone-binding globulin, STS, steroid sulfatase, SULT, sulfotransferase, T, testosterone (androst-4-ene-17β-ol-3-one), UGT, uridine diphosphate-glucuronosyl transferase

## Abstract

Androgens play an important role in metabolic homeostasis and reproductive health in both men and women. Androgen signalling is dependent on androgen receptor activation, mostly by testosterone and 5α-dihydrotestosterone. However, the intracellular or intracrine activation of C_19_ androgen precursors to active androgens in peripheral target tissues of androgen action is of equal importance. Intracrine androgen synthesis is often not reflected by circulating androgens but rather by androgen metabolites and conjugates. In this review we provide an overview of human C_19_ steroid biosynthesis including the production of 11-oxygenated androgens, their transport in circulation and uptake into peripheral tissues. We conceptualise the mechanisms of intracrinology and review the intracrine pathways of activation and inactivation in selected human tissues. The contribution of liver and kidney as organs driving androgen inactivation and renal excretion are also highlighted. Finally, the importance of quantifying androgen metabolites and conjugates to assess intracrine androgen production is discussed.

## Highlights

• Serum concentrations of active androgens do not account for all androgen activity.
• Dysregulation of intracrine androgen pathways is relevant to numerous disease states.
• Androgen metabolites and conjugates can be of diagnostic value.
• 11-oxygenated C_19_ steroids contribute to the androgen pool.
• 11βHSD enzymes are important modulators of both glucocorticoid and androgen activity.

## 1 Introduction

**Fig. 1:**
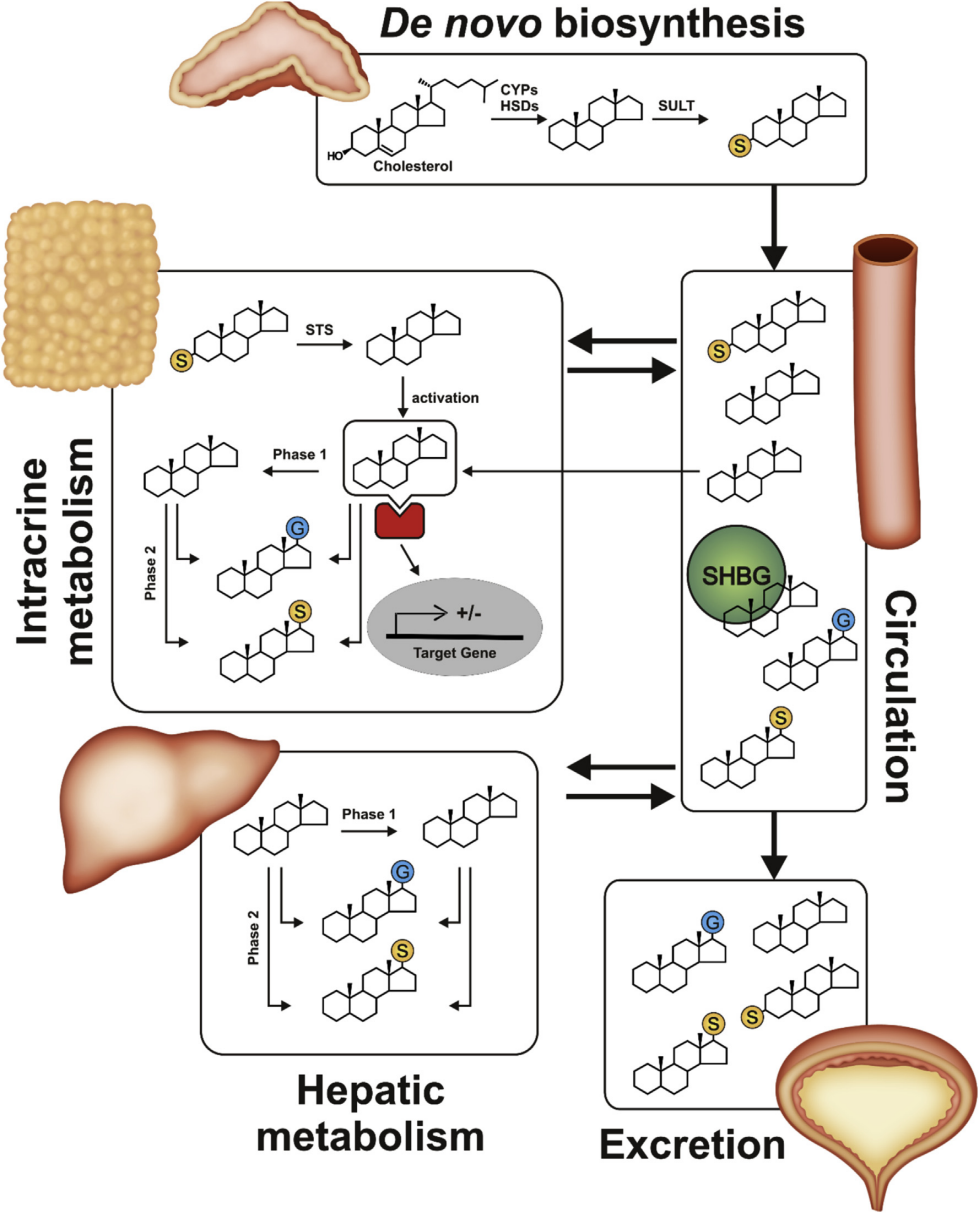
**Schematic overview of C**_**19**_**steroid biosynthesis, intracrine action, metabolism and urinary excretion.** Yellow balls represent the androgen sulfate, blue balls the glucuronide. After metabolic activation the androgen binds the androgen receptor (red) and the complex translocates into the nucleus (grey) to work as transcription factor. It should be noted that while intracrine pathways play the major role for peripheral androgen action, active androgens such as T are produced by the testes and the adrenal, albeit only low levels, and these do not require activation in target tissue. CYP, cytochrome P450; HSD, hydroxysteroid dehydrogenase; SHBG, sex hormone binding globulin; STS, steroid sulfatase; SULT, sulfotransferase.

Androgens (from the greek “*andro”* meaning male or man) are traditionally considered male sex steroids responsible for the maintenance of male characteristics via the activation of the Androgen Receptor (AR), a ligand-induced nuclear receptor that functions as transcription factor after activation. The primary androgen found in men is testosterone (T, androst-4-ene-17β-ol-3-one), which is produced by the Leydig cells of the testes, and released into circulation. The androgen signal can be further amplified in selected target tissues where T is reduced to 5α-dihydrotestosterone (DHT, 5α-androstan-17β-ol-3-one), which is considered the most potent natural androgen (Pretorius et al., 2016, Rege et al., 2013, Storbeck et al., 2013, Wilson and French, 1976). While the adrenal glands only produce low levels of the active androgen T, they produce significant levels of the inactive C_19_ androgen precursors dehydroepiandrosterone (DHEA, androst-5-ene-3βol-17-one) and its sulfate ester DHEAS, androstenedione (A4, androst-4-ene-3,17-dione) and 11β-hydroxyandrostenedione (11OHA4), which in the majority are released into circulation (Rege et al., 2013). After uptake into a peripheral tissue with the required enzyme machinery, these androgen precursors are converted into active androgens which elicit a physiological response. This distinct mechanism of androgen precursor activation, action and inactivation in peripheral androgen-target cells was first termed intracrinology by Labrie et al. (1988) and is linked to classical genomic androgen signalling in both men and women. The importance of intracrinology has been well documented by several authors during the last 25 years after the initial description by Labrie, 1991, Labrie, 2015, Labrie et al., 2001, Labrie et al., 2017) and significant progress has been made in understanding the tissue-specificity of intracrinology and its dysregulation, which is associated with, for example, metabolic dysfunction and hormone dependent cancers. In this review, we give an overview of the journey of C_19_ steroids from their synthesis to their secretion as summarised in Fig. 1. We provide an overview of human C_19_ steroid biosynthesis, the transport of these steroids in circulation and uptake into peripheral target tissues of androgen action. We conceptualise the mechanisms of intracrinology and review the intacrine pathways and effects in selected tissues. The contribution of liver and kidney as organs driving androgen inactivation and excretion are also highlighted. For ease of reference the enzymes discussed in this review are summarised in Table 1 along with the names of the corresponding genes and their enzymatic activities.

**Table 1:** **Summary of enzymes involved in androgen biosynthesis and metabolism**. Genes are named according to HUGO nomenclature (Povey et al., 2001, Shows et al., 1979) (http://www.genenames.org/). In the case of multi-functional enzymes only the major activity towards C_19_ steroids are listed. It should be noted that while hydroxysteroid dehydrogenase enzymes are, in principle, bi-directional enzymes, their directionality *in vivo* (reductive or oxidative) is regulated in part by cellular redox status.

Gene name	Enzyme name and abbreviation	Enzymatic activity towards C_19_ steroids
*AKR1C1*	aldo-keto reductase 1C1, AKR1C1	reductive 3αHSD (minor)
*AKR1C2*	aldo-keto reductase 1C2, AKR1C2	reductive 3αHSD
*AKR1C3*	aldo-keto reductase 1C3, AKR1C3 (also known as 17β-hydroxysteroid dehydrogenase type 5, HSD17B5)	reductive 17βHSD
*AKR1C4*	aldo-keto reductase 1C4, AKR1C4	reductive 3αHSD
*AKR1D1*	aldo-keto-reductase 1D1, 5β-reductase, AKR1D1	5β-reductase
*CYP11A1*	cytochrome P450 cholesterol side-chain cleavage, CYP11A1	C_20_-C_22_ bond cleavage
*CYP11B1*	cytochrome P450 11β-hydroxylase, CYP11B1	11β-hydroxylase
*CYP17A1*	cytochrome P450 17α-hydroxylase/17,20-lyase, CYP17A1	17α-hydroxylation and C_17_-C_20_ bond cleavage
*CYP19A1*	cytochrome P450 aromatase, CYP19A1	C_10_-C_19_ demethylation/A-ring aromatisation
*HSD3B1*	3β-hydroxysteroid dehydrogenase type 1, HSD3B1	oxidative 3βHSD/Δ^5−4^-isomerase
*HSD3B2*	3β-hydroxysteroid dehydrogenase type 2, HSD3B2	oxidative 3βHSD/Δ^5−4^-isomerase
*HSD11B1*	11β-hydroxysteroid dehydrogenase type 1, HSD11B1	(predominantly) reductive 11βHSD
*HSD11B2*	11β-hydroxysteroid dehydrogenase type 2, HSD11B2	oxidative 11βHSD
*HSD17B3*	17β-hydroxysteroid dehydrogenase type 3, HSD17B3	reductive 17βHSD
*HSD17B6*	17β-hydroxysteroid dehydrogenase type 6, HSD17B6 (also known as retinol dehydrogenase, RoDH with *RoDH* used for the gene)	oxidative 3α-HSD
*PAPSS2*	3′-phosphoadenosine 5′-phosphosulfate synthase 2, PAPSS2	3′-phosphoadenosine 5′-phosphosulfate synthase
*SRD5A1*	steroid 5α-reductase type 1, SRD5A1	5α-reductase
*SRD5A2*	steroid 5α-reductase type 2, SRD5A2	5α-reductase
*SRD5A3*	steroid 5α-reductase type 3, SRD5A3	5α-reductase (minor)
*STS*	steroid sulfatase, STS	hydrolysis of steroid sulfates
*SULT2A1*	sulfotransferase 2A1, also DHEA sulfotransferase, SULT2A1	sulfotransferase
*SULT2B1*	sulfotransferase 2B1 isoforms a and b, SULT2B1a and SULT2B1b	sulfotransferase

## 2 *De-novo* androgen biosynthesis in the adrenal and gonads

### 2.1 Adrenal androgen biosynthesis

**Fig. 2:**
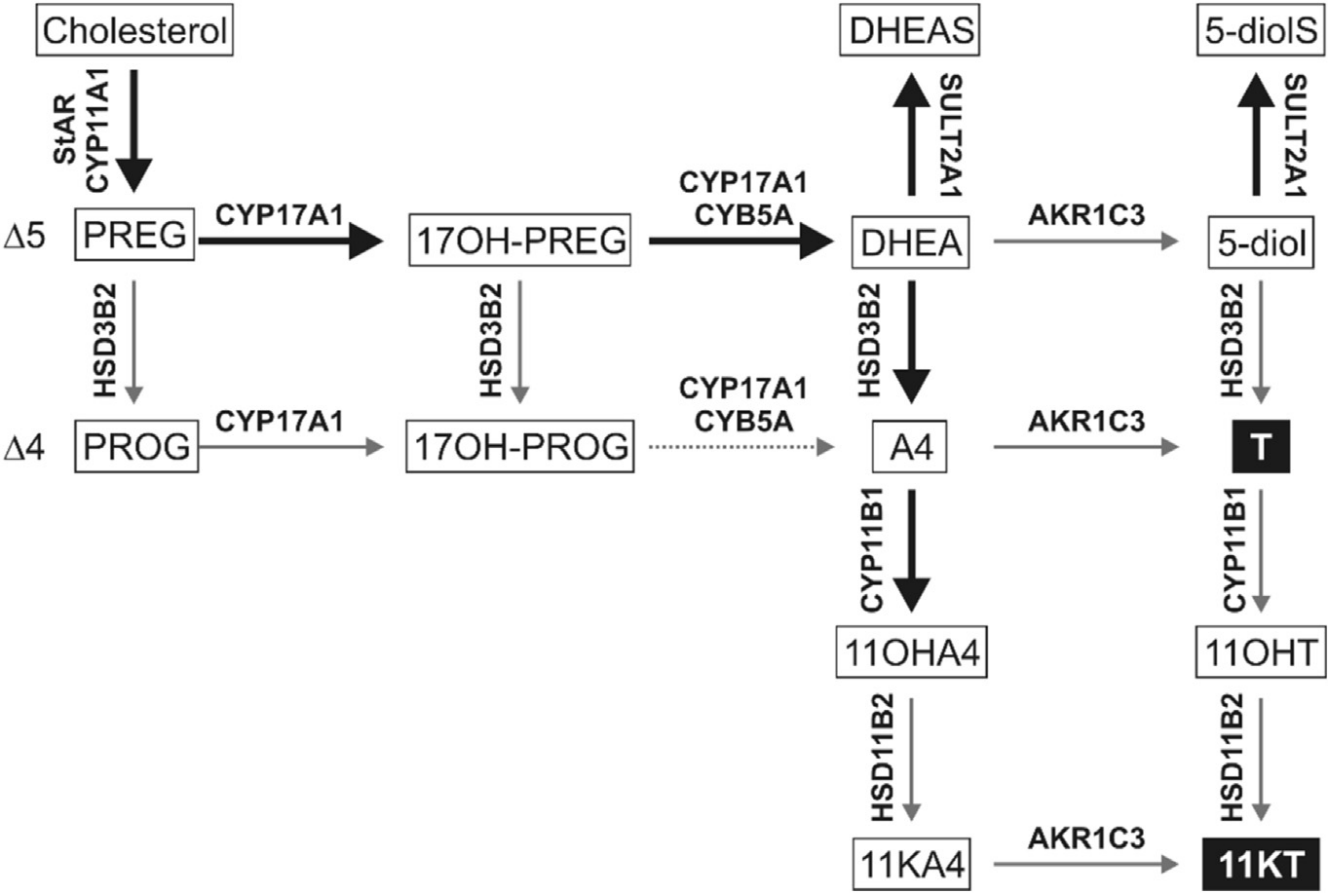
**Schematic overview of C**_**19**_**steroid biosynthesis in the adrenal**. *De-novo* androgen biosynthesis in the *zona reticularis* of the adrenal proceeds via the classical Δ^5^ pathway. CYP11B1 activity leads to the formation of 11-oxygenated C_19_ steroids. Major pathways are indicated by bold arrows and arrows are labelled with the major isoform of the enzyme responsible for the reaction. Active androgens are shown in black boxes.

Adrenal androgen biosynthesis takes place in the *zona reticularis* and proceeds via the classical Δ^5^ pathway (Miller and Auchus, 2011, Turcu and Auchus, 2015) as depicted in Fig. 2. The specific co-localisation of the cytochrome P450 17α-hydroxylase/17,20-lyase (CYP17A1) and cytochrome *b*_5_ in the *zona reticularis* favours the 17,20-lyase activity of CYP17A1 and thus C_19_ steroid production. Furthermore, the expression of *HSD3B2* encoding 3β-hydroxysteroid dehydrogenase/Δ^5−4^-isomerase type 2 (HSD3B2) is relatively low in the *zona reticularis*, thereby ensuring the activity of the Δ^5^ pathway and the production of DHEA.

DHEA is converted to DHEAS by DHEA sulfotransferase (sulfotransferase 2A1, SULT2A1) and as such DHEAS is the major C_19_ steroid secreted by the adrenal, circulating in low micromolar concentrations. Importantly, all sulfotransferases (SULTs) require 3′-phospho-adenosine-5′-phosphosulfate (PAPS), the ubiquitous sulfate donor, which in humans is produced by the two isoforms of PAPS synthase from sulfate and ATP. Mutations of PAPS synthase type 2 (PAPSS2) lead to impaired PAPS synthesis and thus impaired sulfation, which results in enhanced conversion of DHEA to active androgens. Clinically this results in androgen excess manifesting with premature pubarche and a phenotype in women resembling polycystic ovary syndrome (PCOS), also observed in heterozygous carriers of major loss-of-function mutations (Noordam et al., 2009, Oostdijk et al., 2015). In the adrenal, DHEA can further be converted to A4 by HSD3B2 and A4 can be transformed to T by the low adrenal levels of aldo-keto-reductase family 1C3 (AKR1C3, also known as 17β-hydroxysteroid dehydrogensae type 5, HSD17B5), which has 17β-hydroxysteroid dehydrogenase (17βHSD) activity (Nakamura et al., 2009b, Rainey and Nakamura, 2008, Rege et al., 2013). AKR1C3 can also catalyse the conversion of some DHEA to 5-diol (androst-5-ene-3β,17β-diol), which can be sulfated by SULT2A1 or converted to T by HSD3B2. A small fraction of DHEA and 5-diol can also be esterified with fatty acids at the 3β- or 17β-hydroxyl position (Belanger et al., 1990, Hochberg, 1998). Due to the ample levels of cytochrome P450 steroid 11β-hydroxylase (CYP11B1), the adrenal gives rise to 11-oxygenated C_19_ steroids by converting A4 and T to their 11β-hydroxyl derivatives, with 11OHA4 being the major product due to the high abundance of A4. Both 11OHA4 and 11β-hydroxytestosterone (11OHT) may be converted to their 11-keto counterparts by the low levels of 11β-hydroxysteroid dehydrogenase type 2 (HSD11B2) present in the adrenal, though peripheral HSD11B2 may make a more significant contribution (Pretorius et al., 2017, Turcu et al., 2016). Adrenal vein sampling of healthy women has shown that the adrenal secretes DHEAS (low micromolar range) » DHEA, 11OHA4, A4 (medium nanomolar ranges) > 5-diol, 11-ketoandrostenedione (11KA4), T, 11OHT, 11-ketotestosterone (11KT) and DHT (low nanomolar ranges) (Rege et al., 2013). The adrenal thus secretes only very low amounts of active androgens, but mainly androgen precursors that are activated in peripheral tissues.

It should be noted that adrenal C_19_ steroid production is initiated during adrenarche, a pre-pubertal event (approximately age 8 in girls and 9 in boys (Idkowiak et al., 2011)) unique to humans and higher primates (Abbott and Bird, 2009, Arlt et al., 2002, Auchus and Rainey, 2004, Miller, 2008). Adrenarche is due to the development of the *zona reticularis* which is characterised by an increase in the expression of *CYB5A* (encoding cytochrome *b*_5_) and *SULT2A1*, and a decrease in the expression of *HSD3B2*, which in combination favours the biosynthesis of DHEA and DHEAS (Bird, 2012, Gell et al., 1996, Nakamura et al., 2009a). It is only after adrenarche that high levels of C_19_ androgen precursors are available for peripheral activation and adrenarche thus represents the onset of significant intracrine androgen signalling in human life.

A peak of adrenal C_19_ steroid production is reached in the third and fourth decade of human life in both men and women after which levels decline significantly (Labrie et al., 1997b). Nonetheless, 80% of the circulating DHEA in postmenopausal women is of adrenal origin with the remainder produced by the ovary (Labrie et al., 2011).

Importantly, the expression of *CYP17A1* in adrenal of adult mice and rat, which are frequently used as pre-clinical models for endocrine and pharmacology studies, is minimal (Le Goascogne et al., 1991, Mostaghel et al., 2017, Rodriguez et al., 1997). Therefore, unlike humans, mouse and rat adrenals do not produce significant levels of androgen precursors (van Weerden et al., 1992) which can serve as substrates for intracrine action and are therefore not valid models for studies of human androgen intracrinology. Furthermore, while other rodents such as hamster (Cloutier et al., 1995) and guinea pig (Le Goascogne et al., 1991, Tremblay et al., 1995) do produce adrenal C_19_ steroids due to adrenal *CYP17A1* expression, CYP17A1 substrate specificity in these species is significantly different from that of the human enzyme (Cloutier et al., 1997, Tremblay et al., 1995), thus limiting comparisons to human adrenal steroidogenesis. Care should therefore be taken when choosing clinical models for studies of C19 intracrine action.

### 2.2 Ovarian C_19_ steroid biosynthesis

**Fig. 3:**
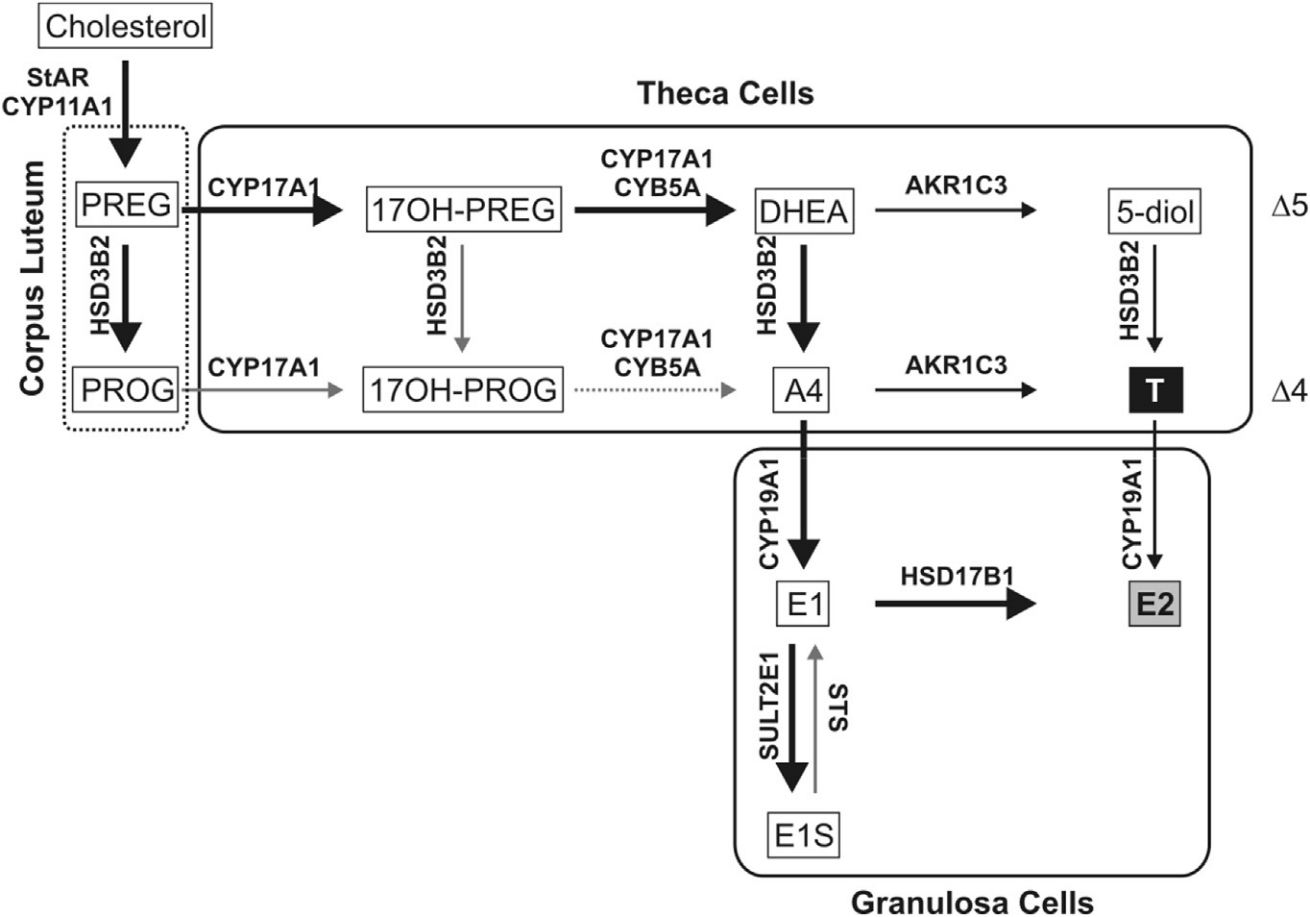
**Schematic overview of sex steroid biosynthesis in the ovaries**. Steps of ovarian *de-novo* sex steroid biosynthesis are partitioned between granulosa and theca cells, which express differential sets of steroidogenic enzymes. Black box: active androgen; grey box: active oestrogen. Besides sex steroids, the ovaries produce high levels of PROG in the corpus luteum after ovulation. Major pathways are indicated by bold arrows and all arrows are labelled with the major isoform of the enzyme responsible for the reaction.

Ovarian steroid biosynthesis can be described by the “two-cell, two-gonadotrophin” model: Two different cell types - granulosa cells of the follicle and the surrounding theca cells - perform distinct reactions due to specific enzyme expression (Fig. 3). In addition, each cell type is differentially regulated by two pituitary hormones – follicle stimulating hormone acting on granulosa cells and luteinising hormone regulating both theca and granulosa cells (Hillier et al., 1994). Granulosa cells do not express *CYP17A1* and their *de-novo* steroidogenic activity therefore stops at the stage of the C_21_ steroids progesterone (PROG) and pregnenolone (PREG) (Voutilainen et al., 1986). These precursors diffuse into the adjacent theca cells, which express *CYP17A1* and *HSD3B2* (but express only low levels of *CYP11A1*), and serve as substrates for the production of A4 (Patel et al., 2010). A4 can either be secreted or converted to T by AKR1C3 in the theca cells (Nelson et al., 2001), but the majority of A4 diffuses back to the granulosa cells where it is converted to estrone (E1), estradiol (E2) and E1-sulfate (E1S) (Miller and Auchus, 2011). 17β-hydroxysteroid dehydrogenase type 1, HSD17B1, is limited to the granulosa cells, where it activates E1 to E2 (Nelson et al., 2001). While the ovary is capable of *de-novo* steroidogenesis, studies have shown that it also efficiently makes use of DHEA of adrenal origin for the production of androgens and oestrogens (Arlt et al., 1999a, Haning et al., 1985, Lebbe et al., 2017). The presence of steroid 5α-reductase type 1 (SRD5A1), aldo-keto reductase 1C2 (AKR1C2), aldo-keto reductase 1C4 (AKR1C4) and 17β-hydroxysteroid dehydrogenase type 6 (HSD17B6) has also been demonstrated for ovarian theca cells and some of those are required for the functioning of the backdoor pathway, which produces DHT by-passing DHEA, A4 and T, (section 5.3), (Marti et al., 2017). Ovarian steroidogenesis commences with puberty when the onset of hypothalamic-pituitary-gonadal signalling leads to an increase in follicle stimulating hormone and luteinizing hormone which in turn regulate steroidogenic activity (Herbison, 2016). While ovarian steroidogenesis demonstrates a menstrual cycle-dependent profile in premenopausal women (Barbieri, 2014), the absence of ovarian follicles in the postmenopausal ovary significantly reduces the production of oestrogens (Labrie et al., 2011). The contribution of postmenopausal ovaries to circulating levels of active androgens is, however, controversial (Couzinet et al., 2001, Fogle et al., 2007).

### 2.3 Testicular C_19_ biosynthesis

**Fig. 4:**
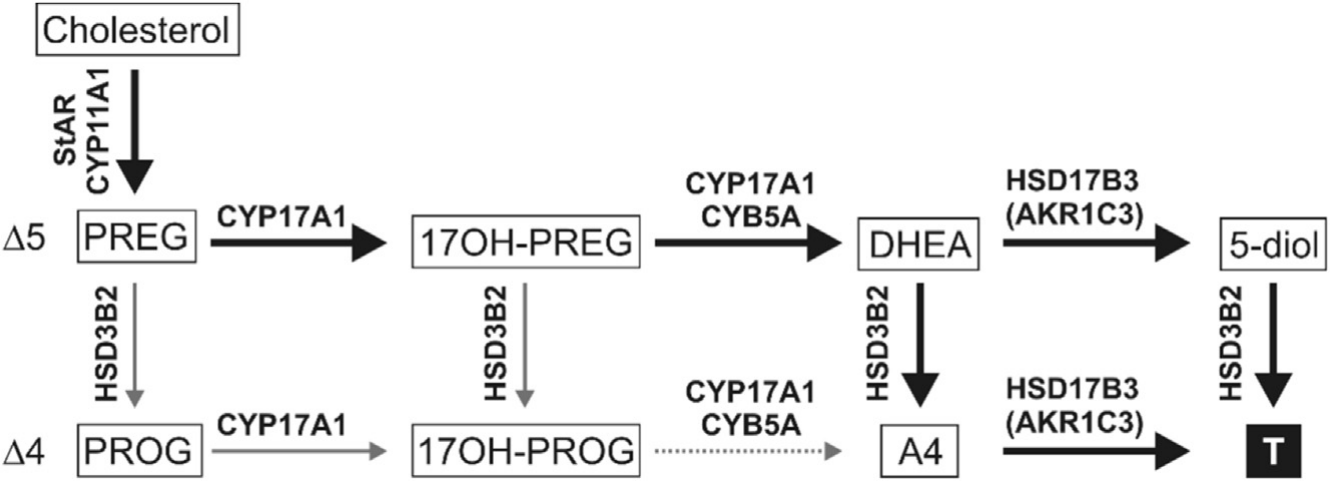
**Schematic overview of C**_**19**_**steroid biosynthesis in the testes**. The testes produce C_19_ steroids via the classical Δ^5^ pathway in the Leydig cells. Due to abundant HSD3B2 and HSD17B3, and the lack of aromatase, T is the major product making the testes the only steroidogenic organ secreting significant amounts of an active androgen (black box). Major pathways are indicated by bold arrows and arrows are labelled with the major isoform of the enzyme responsible for the reaction.

Testicular androgen biosynthesis is carried out in the Leydig cells and, similar to the adrenal, follows the classical Δ^5^ pathway with only minor contribution of the Δ^4^ pathway (Fluck et al., 2003, Sherbet et al., 2003). Due to high abundance of HSD3B2 and 17β-hydroxysteroid dehydrogenase type 3 (HSD17B3) and the absence of SULTs, the final products are the 3-keto-Δ^4^ androgens A4 and T (Fig. 4). HSD17B3 function is essential for testicular T generation from A4 and it is the only human HSD17B isoform with an established deficiency syndrome. HSD17B3 deficiency results in disordered sex development in genetically male children (Boehmer et al., 1999, Mendonca et al., 1999). However, the testes also express *AKR1C3* and in cases of HSD17B3 deficiency the testes still produce low amounts of T via this enzyme (Werner et al., 2012). Concentrations of androgen in testicular venous blood of healthy individuals are T (high nanomolar-low micromolar) » A4, DHEA (medium nanomolar) (Hammond et al., 1977, Ishida et al., 1990, Weinstein et al., 1974).

Unlike the fetal ovaries, the fetal testes have steroidogenic activity which peaks between 11 and 17 weeks of gestation. Fetal testicular T is essential for the development of the male internal genitalia, while the local conversion of testicular T to DHT is essential for the development of external genitalia taking place between 8 and 12 weeks of gestation (Krone et al., 2007, Sobel et al., 2004). Post-natal testicular steroidogenesis is initiated by the onset of hypothalamic-pituitary-gonadal signalling during puberty. A decline of androgens in men starting in the third decade of life can be observed as aging results in a gradual development of testicular failure due to a decreased number of Leydig cells and response to hypothalamic-pituitary signalling (Beattie et al., 2015, Golan et al., 2015). This results in a decline of circulating T concentrations of approximately 0.1 nmol/L per year (Camacho et al., 2013).

## 3 C_19_ steroids in circulation

After production by the adrenal and gonads C_19_ steroids are released into circulation. The concentration of these steroids that peripheral tissue is exposed to is determined by (1) the total concentration of the respective C_19_ steroids in circulation, (2) whether or not the specific steroid is bound to sex hormone binding globulin (SHBG) or albumin and (3) the availability of mechanisms for cellular influx and efflux, although this is only relevant for conjugated steroids which require active transport across the cell membrane (Giorgi and Stein, 1981).

In Table 2, we have summarized the serum concentrations of C_19_ steroids including androgen precursors, active androgens and their metabolites, as determined by liquid chromatography-tandem mass spectrometry in recent studies. In addition to the classical C_19_ steroids (DHEAS, DHEA, T and A4), recent advances in mass spectrometry-based analytical methodology have allowed for the identification and quantification of non-classical androgen metabolites (Bloem et al., 2015). For example, we recently profiled classical and 11-oxygenated androgens in serum of healthy premenopausal women and premenopausal women with PCOS. We found that the 11-oxygenated androgens were the predominant C_19_ steroids in women with PCOS (O'Reilly et al., 2016). Similar results were recently observed by another study which quantified androgen levels in patients with 21-hydroxylase deficiency (Jones et al., 2016, Turcu et al., 2016, Turcu et al., 2017). Significantly, our study in healthy and PCOS women showed for the first time that the circulating levels of the active androgen 11KT were 3–4fold higher than that of T in healthy premenopausal women (O'Reilly et al., 2016), highlighting the important contribution of the 11-oxygenated androgens to the circulating androgen pool in women.

**Table 2:** **Serum concentrations of C**_**19**_**steroids determined by liquid chromatography tandem mass spectrometry**. All concentrations are shown in nmol/L except for DHEA sulfate (DHEAS), which are shown in the micromolar range. The details of the original studies are given in the footnotes below the table.

Androgen precursors	Women	Men	Androgen metabolites	Women	Men
DHEA	4.2–11.8^a^	6.9–30^c^	Androsterone (AST)	0.4–2.1^c^	0.9–1.9^c^
	6.3–35.4^c^	15.8^g^		0.5 ± 0.03^d^	0.7 ± 0.03^d^
	3.4 ± 0.03^d^	4.1 ± 0.1^d^	AST-sulfate	nd^d^	19 ± 1.6^d^
		10–20^j^			1250–2500^j^
DHEA sulfate	3400–9600^a^	1312–14125^c^	AST-glucuronide	28–467^c^	59–248^c^
	701–8965^c^	5709^g^		89 ± 0.7^d^	104 ± 1.4^d^
	3337 ± 16^d^	3247 ± 50^d^		2.1–170^e^	86–150^h^
		4300–5400^j^	EpiAST	0.6 ± 0.03^d^	0.3 ± 0.02^d^
A4	3.3–9.2^a^	2.3–5.5^c^	EpiAST-sulfate	123±3^d^	105 ± 3.2^d^
	1.2–2.82^b^	3.64^g^			500–850^j^
	1.1–8.7^c^		EpiAST-glucuronide	78 ± 0.4^d^	76 ± 1.3^d^
5-androstenediol (5-diol)	1.5 ± 0.07^d^	1.7 ± 0.2^d^	3α-androstanediol (3α-adiol)	nq^d^	0.2 ± 0.02^d^
5-diol-sulfate	215 ± 21^d^	178 ± 10^d^	3α-adiol-sulfate	nd^d^	nd^d^
		250–500^j^	3α-adiol-3-glucuronide	0.6–8.4^c^	1.3–6.7^c^
5α-androstanedione	0.5–2.6^c^	1.0–1.5^c^		0.5–9.2^e^	2.0–3.8^h^
11OHA4	4.9–12.5^a^	2.3–5.1^i^	3α-adiol-17-glucuronide	0.3–10^c^	6.2–8.8^c^
11KA4	2–3.9^a^	0.7–1.4^i^		0.5–12^e^	5.4–11^h^
11OHT	0.1–0.3^a^	0.3–0.7^i^	DHEA-glucuronide	0.9 ± 0.001^d^	0.7 ± 0.03^d^
			7αOH-DHEA	0.08–1.4^f^	
**Active Androgens**			7βOH-DHEA	0.08–0.9^f^	
T	0.2–0.5^a^	7.4–14^c^	7-oxo-DHEA	0.03–0.6^f^	
	0.58–1.1^b^	16.85^g^	16OH-DHEA-sulfate		100–200^j^
	0.4–4.1^c^	15.6 ± 0.6^d^	T-sulfate	nd^d^	nd^d^
	1.1 ± 0.09^d^	10–20^j^	T-glucuronide	0.8 ± 0.02^d^	26.5 ± 0.1^d^
Calculated free T	0.0056–0.0122^b^	0.3780^g^	DHT-sulfate	0.3–2.7^c^	2.5–3.5^c^
DHT	0.2–0.9^c^	0.9–2^c^		nd^d^	nd^d^
	0.4–0.01^d^	1.2 ± 0.09^d^	DHT-glucuronide	nd^d^	nd^d^
11KT	1.2–1.8^a^	1.0–2.6^i^	5-diol-glucuronide	nq^d^	nq^d^

a O'Reilly et al. (2016), interquartile range, n = 49, age interquartile range 23–32.

b Haring et al. (2012), interquartile range, n = 985, age 20-80.

c Trabert et al. (2016), min-max, pre- and postmenopausal women, n = 15.

d Zang et al. (2017), mean ± standard deviation for triplicate of pooled commercial serum.

e Labrie et al. (2006), min-max, pre- and postmenopausal women, n = 424.

f Ke et al. (2016), min-max, pre- and postmenopausal women, n = 34.

g Damgaard-Olesen et al. (2016), geometric mean, n = 72, age 30-<40.

h Vandenput et al. (2007), interquartile range, n = 1086, age interquartile range 18.4–19.3.

i Turcu et al. (2016), interquartile range, combined values for 19 men and 19 women, age 3-59.

j Sanchez-Guijo et al. (2016), interquartile range estimated from Fig. 2, n = 60, age range 18–60.

In both men and women, ageing leads to decreases in C_19_ steroid production (Damgaard-Olesen et al., 2016, Haring et al., 2012, Trabert et al., 2016). However, for women it is unclear if this results from age-related decline of adrenal steroidogenesis or menopause-induced functional alterations of the ovaries or from a combination of both (Labrie et al., 2011, McConnell et al., 1998). In men, the decline can be explained by simultaneous age-related reductions of both adrenal and testicular androgen biosynthesis (Beattie et al., 2015, Camacho et al., 2013, Golan et al., 2015, Labrie et al., 1997b). Although one could easily presume that most C_19_ steroids in circulation are produced by steroidogenic tissues, androgen metabolites released from peripheral tissue make a significant contribution to the circulating pool of C_19_ steroids and thus highlight the importance of local androgen activation and metabolism by intracrine mechanisms. The major fractions of androgen metabolites are conjugated to facilitate their excretion. Indeed, a recent study quantifying androgen metabolites and their sulfo- and glucoconjugates in serum, showed that T and DHT circulate predominantly in their unconjugated form, while their metabolites androsterone (AST, 5α-androstan-3α-ol-17-one), epiandrosterone (5α-androstan-3β-ol-17-one, EpiAST) and 3α-adiol (5α-androstan-3α,17β-diol) were mostly conjugated, each with specific preferences for sulfation or glucuronidation (Zang et al., 2017). It is has previously been suggested that the sum of the circulating metabolites AST-glucuronide and 3α/β-adiol-glucuronide should be used as estimates of active androgens produced in peripheral tissue (Labrie et al., 1997b, Labrie et al., 2003) (3β-adiol, 5α-androstan-3β,17β-diol). Importantly, men and women show comparable concentrations of circulating androgen metabolites (Table 2). Quantification of increased panels of C_19_ steroids including conjugated metabolites are needed to provide further insight into the intracrine metabolism and the role thereof in endocrine disorders.

It should be noted that all C_19_ steroid concentrations discussed above and indicated in Table 2 refer to the total concentrations of androgens in circulation and that this is not necessarily indicative of the steroid concentration available for cellular uptake. The majority of unconjugated C_19_ steroids circulate bound to the plasma proteins albumin or SHBG and only a small fraction (1–2%) circulates in the free form which is accessible to the target tissue. These sex steroid-binding plasma proteins therefore play a crucial role in the regulation of androgen action (Laurent et al., 2016). Although albumin binds all unconjugated steroids with low affinities (μmolar ranges), it makes a significant contribution to steroid binding due to its high abundance (Dunn et al., 1981). In contrast, SHBG binds sex steroids (including active androgens, oestrogens, precursors and metabolites (Avvakumov et al., 2010, Cherkasov et al., 2008, Dunn et al., 1981, Grishkovskaya et al., 2002)) with high specificity and affinity (nanomolar ranges) (reviewed in (Hammond, 2016)). Abnormal levels of SHBG and mutations altering the affinity for its ligands are associated with androgen excess and a PCOS phenotype, but have also been implicated in the pathogenesis of cancer and metabolic dysfunction (Hammond, 2016, Hogeveen et al., 2002).

It is therefore preferable to consider the bioactive fraction in addition to total concentrations when considering the bioactivity of circulating androgens. Several indirect (mathematical) and direct (experimental) approaches can be used (Vermeulen et al., 1999). The “free fraction” (non protein-bound fraction) and the “bioavailable fraction” (unbound and albumin-bound fractions) can be estimated by calculations using total androgen, albumin and SHBG concentrations. The “free androgen index” is defined as (total T*100)/SHBG. Alternatively, free T concentrations can be measured by equilibrium dialysis and the non SHBG-bound fraction can be determined by differential ammonium sulfate precipitation. Free T concentrations usually range around 1–2% of total T and are also age-dependent in women (Haring et al., 2012) and men (Camacho et al., 2013, Damgaard-Olesen et al., 2016). To date, the binding of 11-oxygenated androgens to SHBG and albumin has not been investigated. The fraction of free 11KT relative to that of T is therefore unknown and needs to be determined to gain further insight into the potential physiological role of this potent androgen.

Interestingly, DHEA and 5-diol can be acylated with fatty acids by plasma lecithin:cholesterol acyltransferase located on high density lipoproteins (Jones and James, 1985, Lavallee et al., 1996). The C_19_ steroid fatty acid ester can then be transferred to other lipoproteins. DHEA-fatty acid esters can be taken up by peripheral cells via lipoprotein receptors (Lavallee et al., 1996, Roy and Belanger, 1989). Circulating DHEA-fatty acids have been shown to account for ∼9% of total DHEA in serum (Wang et al., 2011).

## 4 C_19_ steroid metabolism in peripheral target tissues – principles of intracrine androgen activation and inactivation

### 4.1 Cellular uptake and deconjugation

Circulating, bioavailable androgens and their precursors must cross the plasma membrane of target cells to be **(1)** metabolised by enzymes that are located intracellularly in the cytosol or membrane of the endoplasmic reticulum and/or **(2)** to activate the AR, which is localised in the cytosol prior to activation by a suitable ligand. While unconjugated steroids can freely diffuse across the membrane due to their hydrophobic nature, steroid conjugates (sulfates and glucuronides) are hydrophilic and require active transport mechanisms by transmembrane proteins (Giorgi and Stein, 1981). Additionally, de-conjugation is required after influx before the steroid can be metabolised and or interact with its receptor. Organic anion-transporting polypeptides (OATPs) belong to the solute carrier organic anion (SLCO) transporter gene superfamily and are the primary transporters for the influx of conjugated steroids, while multi drug resistant (MDR) proteins belonging to the ATP-binding cassette (ABC) transporters are the primary transporters for the efflux of conjugated steroids (Deeley et al., 2006, Mueller et al., 2015). The access of a conjugated steroid to a specific cell is determined by **(1)** the expression level of transporters in combination with **(2)** the substrate specificity and **(3)** the transport kinetics of the respective transporters. Specific OATPs involved in DHEAS uptake are overexpressed in prostate cancer, leading to an increased intracellular availability of androgen precursors (Wright et al., 2011) and OATP polymorphisms are associated with time to progression during androgen deprivation therapy (Yang et al., 2011). Knock down of OATPs have also been shown to reduce the DHEAS-stimulated proliferation of prostate cancer cell lines (Arakawa et al., 2012). Once transported across the plasma membrane, a steroid sulfate ester is hydrolysed by steroid sulfatase (STS), with maintenance of stereo configuration yielding the respective hydroxysteroid that is subsequently accessible for enzymatic conversions or can exert biological functions (Hobkirk, 1993). STS is a membrane-bound enzyme on the luminal side of the endoplasmic reticulum (Ghosh, 2007) and ubiquitously expressed in all human tissues (Reed et al., 2005). Because of the high concentrations of circulating DHEAS one might assume that STS is a main gate keeper of peripheral androgen metabolism and action. However, while administration of DHEA yields significant increases in both DHEAS and active androgens (Arlt et al., 1998, Arlt et al., 1999b), the administration of DHEAS does not result in any increase of DHEA and downstream androgens in healthy adults (Hammer et al., 2005). By contrast, patients with STS deficiency due to mutation show only a rather mild clinical phenotype with ichthyosis due to the accumulation of sulfated steroids in the skin. They present with a decreased DHEA/DHEAS ratio, which increases to normal levels after puberty, and a slightly increased androgen activation rate as peripheral 5α-reductase activity seems to compensate for the loss of STS function (Idkowiak et al., 2016). STS may therefore rather function as a fine-tuning mechanism for intracellular free steroids. However, STS activity is upregulated in several types of cancer (reviewed in (Mueller et al., 2015)) and has been proposed as drug target in hormone-dependent breast, prostate and endometrial cancers to prevent local oestrogen (including 5-diol, which has oestrogenic effects) and androgen formation from estrone sulfate, DHEAS and 5-diol-sufate. This mechanism has recently been shown to be of relevance also for colon cancer (Gilligan et al., 2017). The potential of STS inhibition has been evaluated in promising clinical trials (Geisler et al., 2011, Purohit and Foster, 2012, Thomas and Potter, 2015).

### 4.2 Principles of intracrine androgen steroid metabolism

After cellular influx, an androgen precursor steroid is enzymatically activated by cell-specific enzymes and pathways before exerting its effect via the AR. Active androgens are subsequently inactivated enzymatically prior to being released from the cell for excretion. This concept of hormone action is termed “intracrinology” and is distinct from the classical concept of “endocrinology” with a designated gland secreting active hormones into circulation exerting direct effects on receptors in target tissues. Intracrinology is defined by the following principles: **(1)** Receptor (AR in case of androgens) and hormone precursors metabolising enzymes are co-expressed in the same cell; **(2)** an inactive hormone precursor (e.g. DHEAS, DHEA, A4, 11OHA4) is taken up from circulation (≠ autocrinology); **(3)** each cell determines the amount of the active hormone (e.g. T, DHT, 11KT, 11KDHT) produced intracellularly by a specific set of enzymes; **(4)** several enzymatic steps are potentially involved in the production of the active hormone and represent different levels of regulation; **(5)** the hormone is enzymatically inactivated in the same cell prior to efflux; **(6)** no significant amount of active hormones are released from the cell into the extracellular space (≠ paracrinology) or circulation (≠ endocrinology) to prevent a systemic excess of active hormones (Labrie, 1991, Labrie et al., 1997a). Significantly, the metabolism of DHEA to active androgens reaches saturation with increasing circulating concentrations of DHEA, thereby protecting peripheral tissues from increased intracellular levels of androgens which may result from pathologically increased biosynthesis of androgen precursors in the adrenal (Labrie et al., 2007). Dysregulation of intracrine pathways can result from **(1)** alterations of expression leading to effects specific for the respective tissue or **(2)** mutations/polymorphisms of involved enzymes leading to systemic effects; and can be associated with metabolic dysfunction or sex steroid-dependent cancer as discussed below.

### 4.3 Enzymes involved in intracrine androgen metabolism

**Fig. 5:**
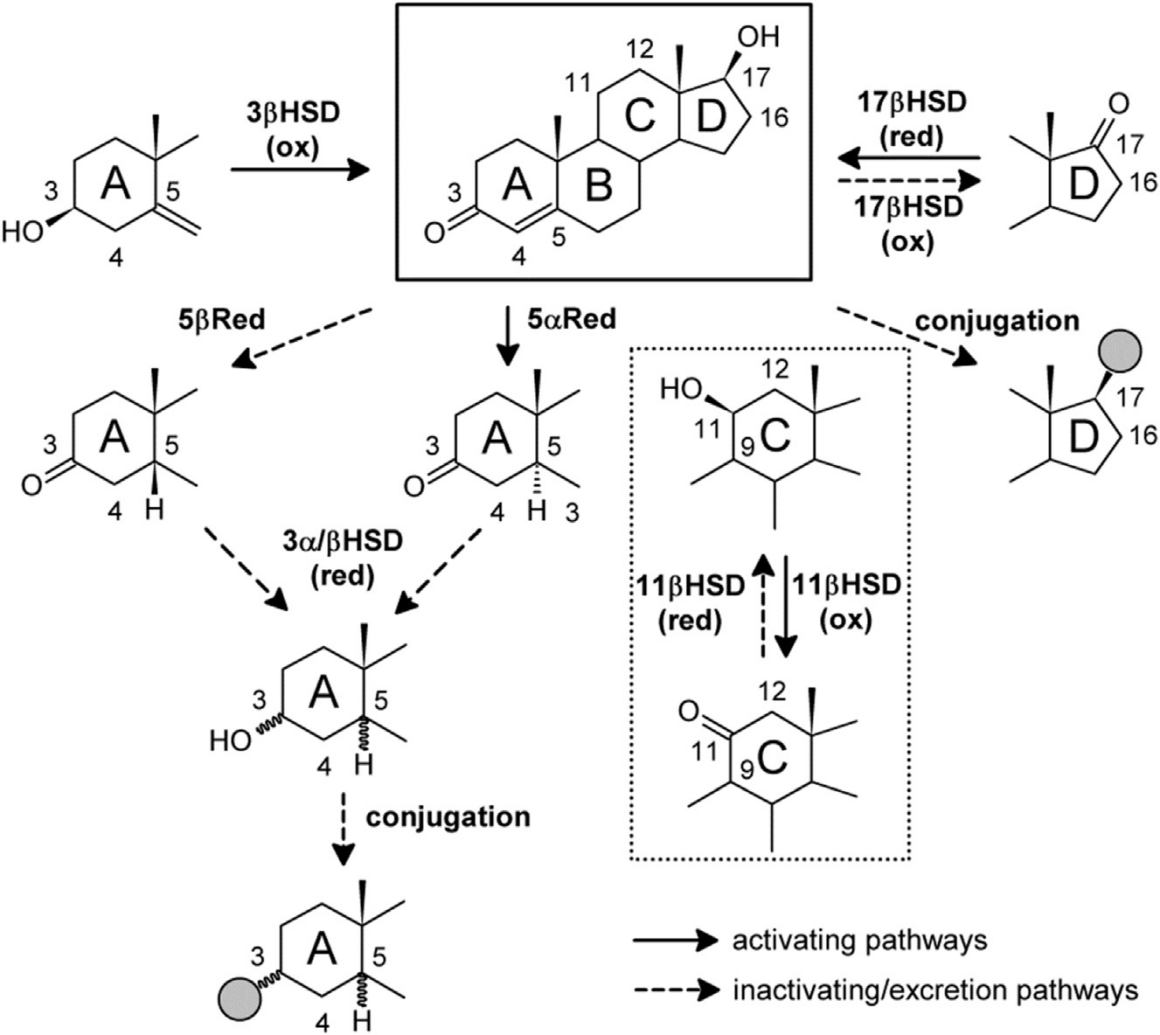
**Principles of androgen activation and inactivation.** All active androgens (T, DHT, 11KT and 11KDHT) share the 3-keto-Δ^4^ and 17β-hydroxyl motifs (shown in the black frame, taking example of T). Solid arrows indicate activation reactions, while dashed arrows represent inactivating reactions and preparation for excretion. 5α-reduction is necessary to achieve maximum AR activation (DHT, 11KDHT). The 11-oxygenated androgens also require the conversion of the 11β-hydroxyl to the 11-ketone in order to obtain maximal activity (11KT and 11KDHT; dotted inset).

Enzymatically catalysed reactions involved in the intracrine metabolism of C_19_ steroids include hydrolysis of sulfate esters, oxidation of the 3β-hydroxyl followed by Δ^5−4^-isomerisation, 11β-oxidation/reduction, 17β-oxidation/reduction, stereoselective 5α- or 5β-reduction of the Δ^4^-double bond, reduction of the 3-keto group, sulfation and glucuronidation of accessible hydroxyl groups. Additionally, C_19_ steroids can serve as substrates for A-ring aromatisation yielding estrogens. HSD3B enzymes introduce the 3-keto-Δ^4^ motif into precursor androgens such as DHEA and 5-diol, while enzymes with reductive 17βHSD function convert the 17-keto group common in androgen precursors to a 17β-hydroxyl group. These two motifs (3-keto-Δ^4^ and 17β-hydroxyl) are shared by all active androgens (Fig. 5). In contrast to the adrenal and gonadal isoform, HSD3B2, which when deficient causes a variant of congenital adrenal hyperplasia, mutations in human *HSD3B1,* which is almost exclusively expressed in peripheral tissues and placenta (Labrie et al., 1992, Simard et al., 2005), are not known, probably as they would prevent placental PROG production during pregnancy (Miller and Auchus, 2011). AKR1C3 (HSD17B5) has been suggested to make the major contribution to peripheral A4 activation by conversion to T (Miller and Auchus, 2011). Certain polymorphisms of *AKR1C3* are associated with PCOS and increased T levels in women (Ju et al., 2015). *AKR1C3* is also overexpressed in prostate cancer, presumably promoting cancer progression by increasing intratumoral androgen levels (Adeniji et al., 2013, Fung et al., 2006).

**Fig. 6:**
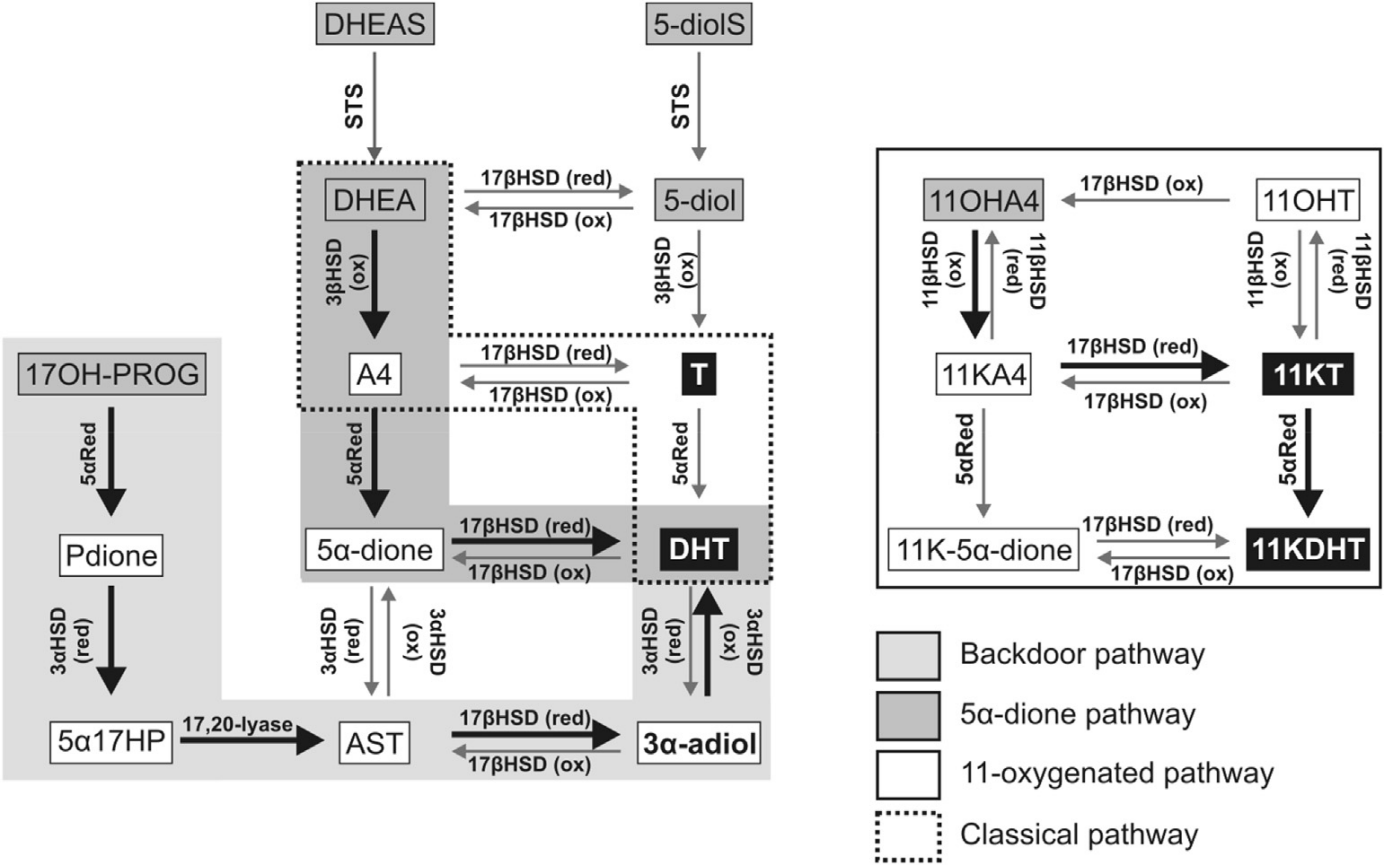
**Schematic of intracrine pathways for the production of active androgens from C**_**19**_**precursors.** Circulating C_19_ steroids (grey boxes) can be converted to active androgens (black boxes) in peripheral tissues exhibiting the required enzymatic activities. Activities are indicated next to each arrow. Pathways of peripheral androgen activation are recurring and distinct from pathways of *de-novo* biosynthesis (Fig. 2, Fig. 3, Fig. 4). 17αHP, 5α-pregnan-3α,17α-diol-20-one; Pdione, 5α-pregnan-17α-ol-3,20-one.

Enzymes with 5α-reductase activity can reduce the Δ^4^-double bond which, in the presence of the 3-keto and 17β-hydroxyl, leads to maximum androgen potential as is observed for DHT and 11KDHT (Pretorius et al., 2016). Systemic upregulation of 5α-reductase activity is observed in women with PCOS leading to enhanced glucocorticoid clearance and enhanced androgen activation in peripheral tissues (Fassnacht et al., 2003, Stewart et al., 1990, Vassiliadi et al., 2009), which is associated with metabolic dysfunction (Conway et al., 2014, O'Reilly et al., 2014b). *SRD5A2* encoding steroid 5α-reductase type 2 (SRD5A2) is expressed in male reproductive tissues (Thigpen et al., 1993) and its disruption leads to the impairment of local DHT formation and in consequence disordered sex development in 46, XY individuals (Okeigwe and Kuohung, 2014, Wilson et al., 1993). The sequence of androgen activation is substrate specific as SRD5A enzymes can also reduce the Δ^4^-double bond of 17-keto steroids prior to their 17β-reduction. Indeed, the conversion of A4 (Δ^4^, 17-keto) to DHT (5α, 17β) proceeds via the 5α-reduction of A4 to 5α-androstanedione (5α-dione, 5α-androstane-3,17-dione), followed by the conversion of 5α-dione to DHT (Chang et al., 2011' Luu-The and Labrie, 2010, Samson et al., 2010) (Fig. 6). Conversely, our group has shown that the activation of 11KA4 (Δ^4^, 17-keto) proceeds by the 17β-reduction to 11KT, followed by 5α-reduction to 11KDHT (5α,17β) (Pretorius et al., 2017) (Fig. 6). It should also be noted that the expression of *HSD11B2*, which encodes the oxidative HSD11B isoform, is required for the activation of 11OHA4, which is the primary 11-oxygenated C_19_ androgen precursor in circulation (Rege et al., 2013, Storbeck et al., 2013, Swart et al., 2013) (Table 2).

Fatty acid esters of androgen precursors can be hydrolysed intracellularly releasing the free steroid and fatty acid acylation of DHEA, 5-diol and T can take place in peripheral tissues, like adipose tissue (Hochberg, 1998, Vihma and Tikkanen, 2011, Wang et al., 2011, Wang et al., 2012).

Following activation, the resulting potent androgens are further metabolised in the target cell of androgen action, yielding inactive metabolites. This metabolism prevents the over-activation of the AR as well as the release of significant amounts of the activated androgens back into circulation (Labrie, 1991). As such the effect of the androgen precursors is maintained within a given target tissue and does not result in a systemic effect (Labrie, 1991, Labrie et al., 1997a). The inactivation reactions can be classified into phase 1 (oxidations and reductions at position 3α/β-, 5α/β, 11β, 17β) and phase 2 metabolism (conjugation of 3/17-hydroxyls), according to the classical two-phase model of detoxification mechanisms (Williams, 1959). The main route of androgen inactivation proceeds **(1)** via the irreversible 5α/β-reduction of the 3-keto-Δ^4^ species, followed by **(2)** the 3α/β-reduction of the 5α/β-reduced androgen. These steps may be followed by **(3)** the conjugation of the emerging 3-hydroxyl or the 17-hydroxyl (Fig. 5). Conjugation can also occur at the 17β-hydroxyl position and can occur prior to 3α/β- and 5α/β-reduction. Both 5α/β-reduction and glucuronidation are irreversible reactions and thus shift the equilibrium towards inactivation.

Unlike 5α-reduction, 5β-reduction inactivates classical AR signalling capability of an androgen. Steroid 5β-reductase is a soluble aldo-keto-reductase enzyme (AKR1D1) and is presumed to be the only human enzyme catalysing the 5β-reduction of C_18_, C_19_, C_21_, and C_27_ 3-keto-Δ^4^ steroids and bile acids (Chen et al., 2011, Chen and Penning, 2014, Kondo et al., 1994). It is mainly expressed in the liver (Charbonneau and The, 2001, Wu et al., 2009) and 5β-reduction therefore plays only a minor role in other tissues. 5α-reduced metabolites are therefore conclusively more indicative for peripheral androgen metabolism than 5β-reduced metabolites, which are more likely to be of hepatic origin (Chen and Penning, 2014).

The 3α- and 3β-reduction of 5α/β -reduced androgens is carried out by members of the AKR1C family, which exert 3α- and 3βHSD activities (Penning et al., 2000, Penning et al., 2004, Steckelbroeck et al., 2004), with different isoforms showing distinct α/β preferences (Steckelbroeck et al., 2004). 5β-reduced androgens are preferably reduced to their 3α-hydroxyl derivative (Jin et al., 2011) making 3β/5β-reduced androgens rare. “Back conversion” of a 3α-reduced androgen to their 3-keto forms (e.g. 3α-adiol to DHT) are possible in presence of an oxidative 3αHSD; 3α- and 3β-hydroxyls can also be interconverted by epimerase activity (Bauman et al., 2006, Belyaeva et al., 2007, Penning et al., 2004).

Androgen metabolites with a 3α/β- and/or 17β-hydroxyl can be conjugated using these hydroxyls for the esterification with either a glucuronosyl group or a sulfate. Conjugation of the 11β-hydroxyl has not been described, thus suggesting that it may not be accessible to the UDP-glucuronosyl transferases (UGT) or SULT enzymes. Glucuronidation of C_19_ steroids is performed by members of the UGT2B subfamily with different substrate preferences and regioselectivity (3 and/or 17), which are expressed in the liver and androgen-sensitive tissues (Belanger et al., 2003). UGTs are microsomal, membrane-bound enzymes catalysing the transfer of the glucuronic acid group of uridine diphospho-glucuronic acid to a functional group (here a hydroxyl) of a specific substrate. As opposed to sulfation, glucuronidation is irreversible and ultimately inactivates the androgen and initiates its renal excretion by increasing polarity and water solubility of the molecule (Belanger et al., 2003). UGT gene variants are associated with serum concentrations of glucuronidated androgen metabolites (Grant et al., 2013) and gene deletions of individual UGTs significantly reduce these concentrations (Nadeau et al., 2011), which is reflected in alterations of urinary T metabolite excretion (Jakobsson et al., 2006). Due to differential expression and regioselectivity of UGTs the comparative study of 3α-adiol-3-glucuronide and 3α-adiol-17-glucuronide can give insight in tissue-specific function of the different UGTs (Barbier and Belanger, 2008, Belanger et al., 2003). As *SULT2A1* expression is mainly limited to the liver, adrenal, colon and kidney (Riches et al., 2009), glucuronidation is the more important conjugation reaction in androgen target tissue. The two SULT2B1 isoforms contribute to androgen metabolite sulfation in prostate, placenta, lung and skin (Falany et al., 2006).

Androgens may also be converted to oestrogens (C_18_ steroids with aromatic A-ring) in tissues expressing *CYP19A1* encoding cytochrome P450 aromatase (CYP19A1). CYP19A1 is a microsomal enzyme catalysing a 3-step C_10_-C_19_ demethylation/A-ring aromatisation of several C_19_ steroids via 19-hydroxy- and 19-aldehyd-intermediates (Beusen et al., 1987, Covey et al., 1987). A4, T, their 16α-hydroxy derivatives and 16βOH-A4 are all substrates for this enzyme (Harada, 1988, Neunzig et al., 2017). Sites of extra-ovarian CYP19A1 expression are placenta, adipose tissue, brain, bone and vasculature (Simpson et al., 2002). The intracrine production of oestrogens from androgen precursors is an essential source of oestrogens in postmenopausal women (Simpson, 2003).

The cell-specific pathways are determined by the respective set of enzymes expressed, their substrate specificities and enzymatic efficiencies, and the intracellular availability of free substrates. Directionality is driven by **(1)** irreversible reactions (5α/β-reduction, P450 oxidation, 3β-hydroxyl oxidation/Δ^5−4^ isomerisation, glucuronidation), **(2)** the relative expression levels of reductive and oxidative HSD enzymes and **(3)** the redox status of the cell which contributes to HSD directionality (Agarwal and Auchus, 2005).

It should again be noted that care should be taken when using animal models to study intracrinology as the enzyme isoforms vary greatly between species. A good example are the significant differences between rodent and human HSD3B and HSD17B enzymes. Rodents have multiple isoforms of HSD3B, while humans have only two. Furthermore, unlike the human enzymes, some rodent HSD3B isoforms have additional 17βHSD activity (de Launoit et al., 1992, Payne et al., 1995, Simard et al., 1995). Similarly, HSD17B enzymes have different isoforms (Marchais-Oberwinkler et al., 2011, Moeller and Adamski, 2009), substrate specificities (Blanchard and Luu-The, 2007, Puranen et al., 1997) and tissue distribution (Martel et al., 1992) in humans and rodents. Enzymes with 3βHSD and 17βHSD activity are also insufficiently characterised in non-human primate models, therefore not allowing for comparisons to human intracrine systems to be drawn.

## 5 Pathways of peripheral androgen activation

### 5.1 The classical androgen biosynthesis pathway

Circulating T, generated from DHEA via A4, can be further activated to DHT in peripheral tissue with 5α-reductase activity. While SRD5A1 catalyses the majority of hepatic 5α-reduction, SRD5A2 in male reproductive tissues (Thigpen et al., 1993) and its deficiency leads to disorders of male sexual differentiation (Wilson et al., 1993). Circulating C_19_ precursors can also be activated by 3βHSD and reductive 17βHSD activity leading to the formation of T. However, the classical pathway via T plays only a minor role in the generation of DHT from inactive C_19_ precursors.

It is worth noting that a third isoform of steroid 5α-reductase (SRD5A3) has been described (Cantagrel et al., 2010, Mitsiades et al., 2012, Uemura et al., 2008) and is expressed in peripheral tissue (Yamana et al., 2010). While the role of SRD5A3 in androgen metabolism is yet to be fully elucidated, SRD5A3 has confirmed polyprenol reductase activity and has been shown to be involved in N-linked glycosylation with S*RD5A3* mutations being linked to congenital disorders of glycosylation (Cantagrel et al., 2010).

### 5.2 The alternate 5α-dione pathway

The alternate 5α-androstanedione pathway bypasses T as intermediate of DHT biosynthesis (Fig. 6). A4 is first 5α-reduced by SRD5A1, yielding 5α-dione due to the higher affinity and catalytic efficiency of SRD5A1 for A4 than for T (Andersson and Russell, 1990, Russell and Wilson, 1994, Sugimoto et al., 1995), which is followed by the conversion of 5α-dione to DHT by reduction of the 17-ketone (Chang et al., 2011, Luu-The and Labrie, 2010, Samson et al., 2010). This pathway is not present in the adrenal or gonads, but plays the major role for peripheral DHT generation from circulating precursors other than T (Luu-The and Labrie, 2010). It has especially been shown to be an essential pathway for intratumoral DHT production from adrenal androgen precursors in CRPC when circulating levels of T are significantly reduced by physical or chemical castration (Chang et al., 2011, Sharifi and Auchus, 2012).

### 5.3 The backdoor pathway

Another alternative pathway to DHT is the so-called “backdoor” pathway which by-passes T, A4 and DHEA. In this pathway, C_21_ precursors, mainly 17α-hydroxyprogesterone (17OHPROG) but also progesterone, are substrates for **(1)** 5α-reductase activity and **(2)** a reductive 3αHSD activity. This leads to generation of 5α17-hydroxypregnanolone (5α17HP; 5α-pregnan-3α,17α-diol-20-one) in the case of 17OHPROG, which then **(3)** undergoes the 17,20-lyase reaction to androsterone (AST) (Auchus, 2004). Reductive 17βHSD activity **(4)** and oxidative 3αHSD activity **(5)** subsequently lead to the conversion of AST to DHT (Fig. 6). The initial 5α- and 3α-reductions support the C_17_-C_20_ side-chain cleavage by delivering 5α17HP, which is the best substrate for the 17,20-lyase activity of CYP17A1 and whose cleavage is not dependent on cytochrome *b*_5_ (Gupta et al., 2003). In humans, the backdoor pathway is relevant in pathological conditions when 17OHPROG accumulates, such as in congenital adrenal hyperplasia due to cytochrome P450 oxidoreductase deficiency (PORD) or 21-hydroxylase deficiency (Arlt et al., 2004, Homma et al., 2006, Kamrath et al., 2012, Krone et al., 2012, Miller and Auchus, 2011). In PORD, the alternative pathway can result in sufficient virilisation of affected 46, XY individuals in the prenatal period, despite the complete disruption of the classic androgen pathway, as CYP17A1 will still convert 5α17HP towards DHT but no longer 17α-hydroxy-pregnenolone and 17OHPROG to DHEA and A4, respectively.

### 5.4 The 11-oxygenated androgen pathways

Pathways for the generation of active 11-oxygenated androgens (11KT and 11KDHT) start with the CYP11B1 catalysed 11β-hydroxylation of A4 and T (Swart et al., 2013). The adrenal is the only source for 11-oxygenated C_19_ precursors due to the adrenal specific expression of CYP11B1. It is not surprising that 11OHA4 is the predominant 11-oxygenated C_19_ steroid produced as the adrenal produces significantly more A4 than T (Rege et al., 2013). Both 11OHA4 and 11OHT are released into circulation and can subsequently be activated in peripheral tissues (Fig. 6). Both steroids can be converted to their respective 11-keto forms by HSD11B2. While 11OHA4 is not a substrate for reduction of the 17β-hydroxyl, 11KA4 is readily converted to 11KT which can be 5α-reduced to 11KDHT (Bloem et al., 2013, Pretorius et al., 2017, Swart and Storbeck, 2015). While 11OHT and 11OHDHT represent partial AR agonists, 11KT and 11KDHT are full agonists with the same AR activating potential as T and DHT, respectively (Pretorius et al., 2016, Storbeck et al., 2013). The presence of the reductive enzyme HSD11B1, in some peripheral tissues may therefore prevent the activation of 11OHA4 and catalyse the inactivation of 11KA4 and 11KT, which are also in circulation, albeit at significantly lower levels than 11OHA4 (O'Reilly et al., 2016). The relative activities of HSD11B2 and HSD11B1 are therefore critical in determining the activity of 11-oxygenated androgens in peripheral tissue. This additional level of regulation suggests that the 11-oxygenated androgens have a more select number of target tissues in comparison to the classical androgens.

## 6 Directionality of biosynthetic steps, phase 1 metabolism and conjugation

The classical concepts of androgen biosynthesis and metabolism suggest that conjugation is the final step of a pathway. For example, DHEA is sulfated by SULT2A1 to DHEAS at the end of adrenal androgen biosynthesis, and detoxification is achieved by sequential phase 1 and 2 metabolism; with the phase 1 reaction sometimes even being required to allow a phase 2-reaction, e.g. the reduction of the 3-ketone to give a hydroxyl accessible for conjugation. However, increasing evidence suggests that this directionality is not obligate.

A small number of enzymatic pathways have been described that can directly interconvert sulfated steroids and proceed analogously to the biosynthetic pathways of free androgens. DHEAS has been shown to be converted to 5-diol-3β-sulfate by 17βHSD activity present in human testes (Ruokonen, 1978) and T-sulfate has been shown to be converted to estradiol-17-sulfate by CYP19A1 in human placental microsomes (Satoh et al., 1992). Furthermore, CYP17A1 has been shown to be able to 17α-hydroxylate PREG-sulfate in a recombinant human cell line, but could not catalyse the subsequent 17,20-lyase reaction (Neunzig et al., 2014). Interestingly, recombinant HSD3B2 has been shown to have steroid sulfatase activity converting PREG-sulfate to PREG (Sanchez-Guijo et al., 2016). Although a physiological meaning of these reactions has not been established, their discovery clearly shows an underestimated role of sulfated steroids in steroid metabolism and action.

Interestingly, phase 2 reactions can precede phase 1 reactions during androgen inactivation. After glucuronidation and sulfation androgen metabolites can be metabolised by AKR1C subfamily members, e.g. DHT-17-glucuronide/sulfate to 3α-adiol-17-glucuronide/sulfate with kinetic parameters even indicating a preference of the 17-glucuronidation preceding the 3α-reduction (Jin et al., 2009, Penning et al., 2010). Finally, foetal CYP3A7 has been shown to catalyse the 16α-hydroxylation of DHEAS (Ohmori et al., 1998).

## 7 Intracrinology in metabolic target tissues

### 7.1 Adipose tissue

Adipose tissue expresses isoforms of all enzymes required for the activation of androgens from circulating precursors (STS, HSD3B1, reductive 17βHSDs, namely HSD17B1, HSR17B3 and AKR1C3 with involvement in androgen activation, and SRD5A1) as well as their subsequent inactivation (reductive 3αHSDs (mainly AKR1C2), UGT2B15 and UGT2B17) (Blouin et al., 2009b, O'Reilly et al., 2014a, Tchernof et al., 2015). The activation of androgens within adipocytes has been shown to regulate proliferation and differentiation, insulin sensitivity, adipokine signalling and lipid metabolism (O'Reilly et al., 2014a). While functional studies of androgen conversion within adipocytes are fragmentary, there is evidence for the importance of HSDs from the AKR1C subfamily. *AKR1C1* (predominant 20αHSD with low reductive 3αHSD activity), *AKR1C2* (reductive 3αHSD activity) and *AKR1C3* (reductive 17βHSD activity) show the highest expression levels of all steroid converting enzymes expressed in adipocytes of women and men (Blouin et al., 2009b) and their expression and activity correlates with obesity in women and men (Blouin et al., 2005, Blouin et al., 2006). *AKR1C3* expression has been shown to decrease with weight loss in female subcutaneous adipose tissue (Quinkler et al., 2004). AKR1C3 (reductive 17βHSD, A4→T) and AKR1C2 (reductive 3αHSD, DHT→3α-adiol) activity has been shown for adipocytes from both men and women with higher activity of the inactivating 3αHSD (Blouin et al., 2003, Blouin et al., 2006). The correlation of both activating and inactivating enzymes with obesity indicates an increased local production and metabolic clearance of androgens highlighting the importance of adipose tissue intracrinology in obesity. Measurements of sex steroid levels in adipose tissue of obese men (ng/g) revealed general levels of DHEA > **A4** ≥ **T** > E1 > DHT with differences between omental and subcutaneous depots (Belanger et al., 2006). The approximation of A4 and T levels compared to circulating concentrations highlights the importance of reductive 17βHSD activity (AKR1C3) converting A4 to T local androgen load (Belanger et al., 2006). Importantly, AKR1C3 expression and activity increases with the differentiation of preadipocytes to mature adipocytes and the interconversion of A4 and T is shifted in favour of T generation only in mature adipocytes (Quinkler et al., 2004). The expression of UGTs in adipose tissue (Tchernof et al., 1999) and the correlation of plasma 3α-adiol glucuronide with fat mass in men (Tchernof et al., 1997) suggest phase 2 metabolism as final step of androgen inactivation in adipocytes.

Androgen excess is recognised as the major determinant driving metabolic dysfunction observed in women with PCOS (Conway et al., 2014) and the degree of androgen excess correlates with the severity of insulin resistance in PCOS (O'Reilly et al., 2014b). We have recently shown that serum T levels correlated with BMI in both healthy women and women with PCOS. This data supports the AKR1C3 catalysed conversion of A4 to T within adipose tissue and further suggests that not all T produced within adipocytes is inactivated, but that a portion of the T is also released in circulation. Interestingly, we also showed that in the same cohort 11KT did not correlate with BMI, which is not surprising given that HSD11B1 is present in adipose tissue, and not HSD11B2. This prevents the conversion of 11OHA4 to 11KA4, which is a prerequisite for the subsequent conversion of 11KA4 to 11KT by AKR1C3 (O'Reilly et al., 2016).

Aromatisation of androgens to oestrogens takes place in male and female adipose tissue (Longcope et al., 1978, McTernan et al., 2000) and increased aromatization has been proposed as a major mechanism leading to obesity-induced male androgen deficiency (Cohen, 1999). The expression of aromatase in adipose tissue has also been shown to be elevated in postmenopausal women (Bulun and Simpson, 1994) and as such adipose tissue is an important source of oestrogen in these women (Simpson, 2003). The effects of androgens on adipocyte function and the expression pattern of metabolizing enzymes are defined by sex, fat depot localisation and menopause status (Blouin et al., 2009a, O'Reilly et al., 2014a, Quinkler et al., 2004).

### 7.2 Skeletal muscle

Androgens exhibit beneficial effects on skeletal muscle function supporting myogenic differentiation and improving protein synthesis, lipid oxidation, insulin sensitivity, glucose utilisation and mitochondrial function (Kelly and Jones, 2015). Furthermore, muscle regeneration and recovery is supported by the androgen-dependent regulation of muscle satellite cells (MacKrell et al., 2015). As a consequence of their higher circulating T levels, men have higher lean mass then women (Wells, 2007). Muscle cells exhibit 3βHSD, reductive 17βHSD and 5α-reductase activity resulting in the activation of DHEA to T and DHT. Expression decreases during ageing and can be rescued by resistance training (Sato et al., 2014a, Sato et al., 2014b). The production of T from A4 by AKR1C3 has been proposed to be the essential step of androgen activation in muscle (Lin et al., 1997, Longcope and Fineberg, 1985, Luu-The and Labrie, 2010), with the further conversion of T to DHT seeming to play only a minor role as there is no clarity regarding the expression of *SRD5A* isoforms (Luu-The and Labrie, 2010). A4 and T can also be aromatised in skeletal muscle (Longcope et al., 1978, Matsumine et al., 1986), with low activity and expression of aromatase. However, as skeletal muscle represents a major part of the human body, its contribution to systemic oestrogen levels has been proposed to be significant in men and post-menopausal women (Larionov et al., 2003). 3α-reduction and phase 2 metabolism in skeletal muscle has not been studied to date and the contribution of skeletal muscle to circulating levels of active androgens cannot conclusively be ruled out at present. No mRNA of *SULT2A1* or *SULT2B1* has been detected in skeletal muscle (He et al., 2005, Luu-The et al., 1995).

### 7.3 Liver

#### 7.3.1 Hepatic phase 1 metabolism

**Fig. 7:**
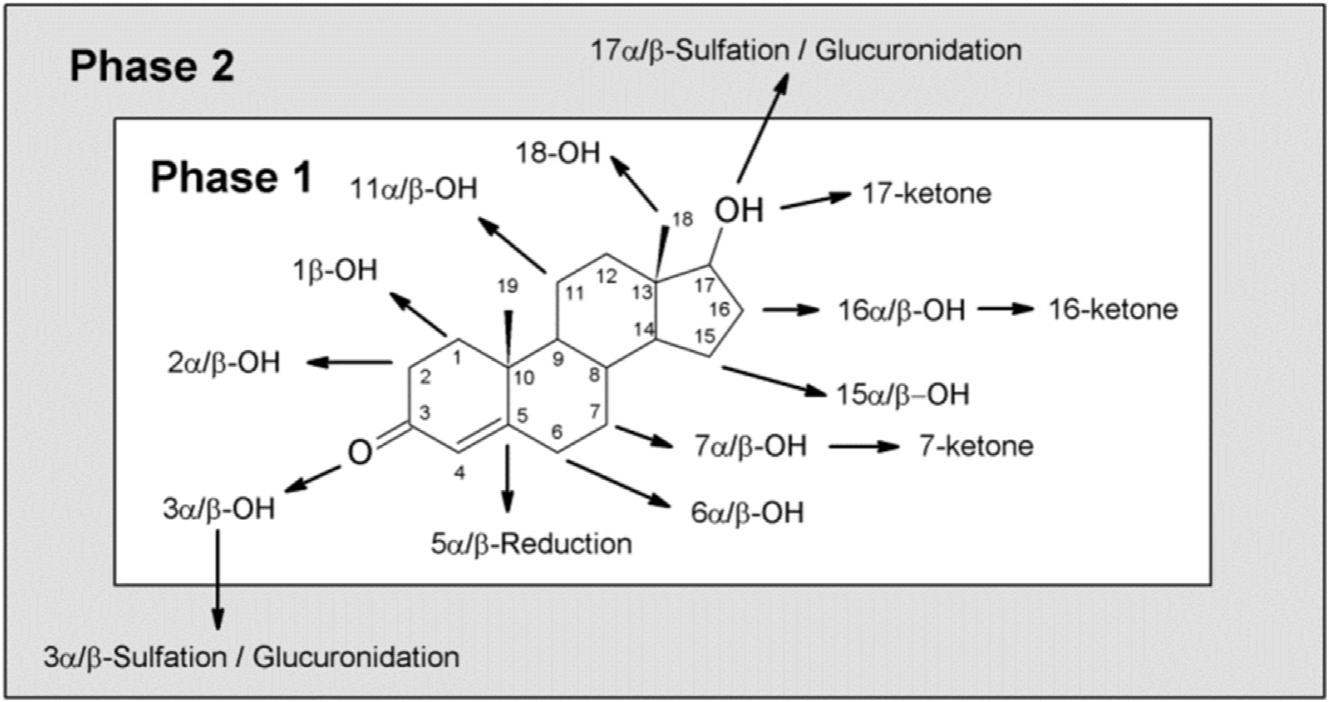
**Overview of hepatic phase 1 and 2 metabolism of C**_**19**_**steroids.** Phase 1 metabolism comprises the 5α/β-reduction of the Δ^4^ double bond and the reduction of the 3-keto group to its 3α/β-hydroxyl as well as hydroxylations and further oxidations at various positions. Phase 2 reactions consist of the conjugation of the 3- and 17-hydroxyls. 17α-conjugates originate from Epitestosterone. The reactions shown in the figure summarize those described for T, A4 and DHEA.

The liver catalyses extensive phase 1 and phase 2 metabolism of xenobiotics and endogenous hormones including steroids, thereby regulating their activity and clearance. Hepatic phase 1 metabolism of androgens includes the following types of reactions: **(1)** 5α/β-reduction of the Δ^4^-double bond followed by 3α/β-reduction of the 3-ketone; **(2)** oxidations by a large set of hepatic P450 enzymes; **(3)** HSD reactions of 11β- and 17β-hydroxyls if available and of hydroxyls introduced by P450s. Fig. 7 provides a summary of reactions catalysed by drug- and xenobiotic-metabolising liver enzymes.

The liver has high levels of the 5β-reductase AKR1D1 and primarily produces 5β-reduced androgens (e.g. etiochonanolone, 5β-androstan-3α-ol-17-one, ETIO) in contrast to peripheral tissue which produce 5α-reduced species (Charbonneau and The, 2001, Chen and Penning, 2014, Penning, 2010). The subsequent formation of the 3α-reduced counterpart of a 5β-reduced steroid is favoured over the 3β-reduction leading to mainly 3αOH-5β-reduced metabolites (Jin et al., 2011). Mutations of *AKR1D1* lead to severely reduced to completely absent urinary 5β-reduced steroid excretion and hepatic failure, as AKR1D1 is essentially involved in hepatic bile acid synthesis, but no other clinical manifestations (Gonzales et al., 2004, Lemonde et al., 2003, Palermo et al., 2008).

Hepatic phase 1 metabolism is extremely diverse due to the contribution of various P450 enzymes. In contrast to the steroidogenic P450s expressed in adrenals and gonads, hepatic CYPs have a high degree of functional plasticity on different levels: (1) genetic variation (polymorphisms, copy number, promoter variants); (2) variation of expression levels due to inducibility by e.g. xenobiotics; (3) variation of activity (broad substrate specificity and thus competition of different substrates, limited selectivity, multiple substrate binding sites positively or negatively influencing the active site, influence of the allosteric modulator cytochrome *b*_5_ and competition for their electron transfer protein cytochrome P450 reductase) (Zanger and Schwab, 2013). This leads to high inter- and intra-individual variability in hepatic P450 enzyme activity. About a dozen P450s of the CYP1, CYP2, CYP3 and CYP7 families can catalyse hydroxylations with diverse regio- and stereochemistry as depicted in Fig. 7 and summarized in a review by Niwa et al. (2015). Some of these P450s may also catalyse an additional oxidation of a hydroxyl to its ketone (e.g T → A4 or 16βOH-T → 16-keto-T). CYP3A4 is the most abundant P450 in the liver (∼30%) (Shimada et al., 1994) and predominantly, though not exclusively, catalyses the 6β-hydroxylation of T and the 16α- and 7α-hydroxylation of DHEA (Niwa et al., 2015).

Several HSD17B isoforms are active in the liver (Moeller and Adamski, 2009) as well as HSD11B1, which oxidises 11-keto-steroids to their 11β-hydroxyl form. HSD11B1 plays an important role in the recycling of cortisol from its inactive metabolite cortisone (Gathercole et al., 2013) and might therefore also be involved in the hepatic metabolism of 11-oxygenated androgens. HSD11B1 is also involved in the metabolism of the DHEA metabolite 7αOH-DHEA (produced by CYP3A4 and CYP7B1) (El Kihel, 2012). It catalyses the interconversion of 7α- and 7β-OH-DHEA via the 7-keto intermediate (Muller et al., 2006).

Unusual reactions leading to new T metabolites have recently been described and include the methylation of an unknown T metabolite, probably at a hydroxyl functional group, and the de-methylation to C_19_-nor-androgens (Piper et al., 2016). The excretion of 19-nor-AST has previously been described (Dehennin et al., 1999). De-methylation may result from uncomplete C_10, 19_-lyase/A-ring aromatisation reaction by CYP19A1.

#### 7.3.2 Hepatic phase 2 metabolism

Hepatic phase 2 metabolism includes glucuronidation and sulfation. All UGTs capable of androgen glucuronidation are expressed at high levels in the liver (Belanger et al., 2003) leading to intensive hepatic glucuronidation compared to other tissues (Rittmaster et al., 1993). Conjugated metabolites are released from liver cells into circulation by active transport. The main androgen metabolites in circulation derived from hepatic and other peripheral metabolism are 3α-adiol-3-glucuronide, 3α-adiol-17-glucuronide, AST-glucuronide and ETIO-glucuronide with levels higher than for T. Interestingly, the concentration of 17-keto metabolites in circulation is higher than that of the 17β-hydroxy metabolites with AST-glucuronide and -sulfate being the most abundant (see (Kalogera et al., 2013) for a review of LC-MS/MS based studies of circulating androgen and their glucuronides).

Genetic variations of UGTs can change the glucuronidation efficiency for an androgen and alter its excretion (Piper et al., 2016). The formation of androgen linked di-glucuronides (second glucuronidation occurs on the first glucuronosyl moiety) is also possible (Murai et al., 2005, Murai et al., 2006). Discrete di-glucuronidation (conjugation at two different functional groups of the same molecule) has to our knowledge not been described. Steroids are preferentially glucuronidated in either 3- or 17-position depending on the substrate and regio selectivity of the individual UGTs.

The liver also shows high SULT expression and activity compared to other tissues, including *SULT2A1* and *SULT2B1* involved in androgen metabolism (Meloche and Falany, 2001, Riches et al., 2009). The formation of distinct di-sulfates is described for the synthetic androgen tibolone (Falany et al., 2004) and cholesterol (Cook et al., 2009). 16α-hydroxy-DHEAS, 5-diol-sulfate, AST-sulfate, EpiAST-sulfate can be measured in nM concentrations in serum of both men and women (Labrie et al., 1997b, Sanchez-Guijo et al., 2015, Zang et al., 2017). Importantly, women produce androgen metabolites in concentrations comparable to that of men (Labrie et al., 1997b, Trabert et al., 2016, Zang et al., 2017).

#### 7.3.3 The liver as a target tissue of androgen action

Although the liver is traditionally considered only in terms of phase 1 and phase 2 metabolism, the liver also expresses AR turning it into a target tissue for classical androgen action. Both T and DHT have recently been shown to increase lipogenesis in human hepatocytes of female (but not male) donors (Nasiri et al., 2015) and androgens have been shown to play a crucial role in the development of hepatocellular carcinoma (Kalra et al., 2008, Kanda et al., 2014).

## 8 Intracrine androgen metabolism in reproductive target tissues

### 8.1 Breast and endometrium

While oestrogens have essential proliferative effects on the normal mammary gland and breast cancer, androgens and the expression of the AR have demonstrated both positive and negative outcomes in breast cancer as reviewed in (Hickey et al., 2012, McNamara and Sasano, 2015a, McNamara and Sasano, 2016). Both androgens and oestrogens are synthesised from circulating androgen precursors in breast tissue and the homeostasis of these pathways determines health status (McNamara and Sasano, 2015b). Aromatase inhibition is a mainstay of postmenopausal breast cancer treatment and an impressive model for the translation of intracrine modulation to patient care. Androgen metabolism in breast is however, frequently overlooked. Numerous studies have shown that androgen levels are higher within breast tissue than in circulation and are generally higher in benign tissue than cancerous tissue, demonstrating the relevance of intracrinology in breast and its dysregulation in cancer (Stanczyk et al., 2015). It should also be noted that menopausal status significantly influences the intracrinology of breast tissue (McNamara and Sasano, 2015b). Breast cancer tissue has STS, 3βHSD, reductive 17βHSD, 5α-reductase and aromatase activities (Labrie et al., 2003, McNamara and Sasano, 2015b, Suzuki et al., 2005) as well as 3αHSD, UGT, and SULT activity (McNamara et al., 2013). Selective inhibition of HSD3B1 has been shown to slow down proliferation of breast cancer cell line and might just represent an interesting drug target simultaneously tackling androgen and oestrogen synthesis (Thomas et al., 2011). However, as with the heterogeneous expression of steroid receptors within breast cancer the expression of steroid metabolising enzymes is also heterogeneous and the specific pathways followed can therefore vary greatly. The detailed intracrinology of breast tissue is therefore a complex topic and beyond the scope of this review for review see (Africander and Storbeck, 2017, Capper et al., 2016, Labrie et al., 2003, McNamara et al., 2013, McNamara and Sasano, 2015b).

In the endometrium, intracrine production of androgens and estrogens may be involved in regulating decidualisation (transformation of endometrial stromal cells to secretory cells) and receptivity. While aromatase activity increases during decidualisation leading to an increased estrogen secretion (Gibson et al., 2013), time-dependent changes in *SDR5A1* and *AKR1C3* expression alter T and DHT secretion. While T production increases and stays elevated during decidualisation due to constantly increased AKR1C3 levels, DHT secretion initially increases, but then decreases with the progression of the decidualisation process following reductions in SRD5A1 levels (Gibson et al., 2016).

### 8.2 Prostate

Androgens and in particular the intraprostatic conversion of circulating T to DHT by SRD5A2 (Russell and Wilson, 1994) are required for normal prostate development and function (Andersson et al., 1991, Imperato-McGinley et al., 1974). Androgen deprivation therapy in the form of chemical or physical castration is therefore a preferred treatment of advanced prostate cancer. Despite initially demonstrating excellent results the prostate cancer often remerges as castration resistant prostate cancer, which in most cases remains androgen dependent. While the contribution of *de-novo* intratumoral biosynthesis to local androgens has mostly been excluded (Hofland et al., 2010), numerous studies have shown that CRPC is dependent on the intracrine conversion of circulating androgen precursors of adrenal origin to active androgens reviewed in (Capper et al., 2016, Luu-The et al., 2008, Pretorius et al., 2017, Sharifi and Auchus, 2012). Specifically, the alternate 5α-dione pathway catalyses the conversion of DHEA and A4 to DHT while bypassing T completely (Fig. 6). The flux through this pathway is due to the preference of SRD5A1, the dominant isoform expressed in CRPC, for A4 over T, coupled to the poor efficiency by which AKR1C3 converts A4 to T (Chang et al., 2011, Sharifi, 2012). Additionally, the back conversion of 3α-adiol to DHT by oxidative 3αHSDs has been demonstrated for prostate cancer cell lines enabling a recycling of this DHT metabolite (Bauman et al., 2006, Mohler et al., 2011, Rizner et al., 2003). Prostate cells also express CYP3A4 that can inactivate T by conversion to mainly 6βOH-T (with 2β-, 15α/β- and 11β-hydroxyl side product formation (Niwa et al., 2015)) and a decrease of *CYP3A4* expression is observed in prostate cancer (Fujimura et al., 2009). After 5- and 3-reduction androgens are extensively glucuronidated in the prostate producing 3α-adiol-17-glucuronide and AST-3-glucuronide. Genetic variations of UGTs significantly contribute to prostate cancer risk and progression and there is evidence for a down-regulation of UGTs in prostate cancer promoting intratumoral androgen accumulation (Barbier and Belanger, 2008, Gauthier-Landry et al., 2015). Because of the ability of prostate cancer cells to convert 3α-adiol back to DHT its glucuronidation is of particular importance for androgen inactivation and the regulation of cancer progression (Chouinard et al., 2007).

Recent studies have also shown that prostate cancer cell lines are able to metabolise the adrenal androgen precursor 11OHA4 (Storbeck et al., 2013, Swart et al., 2013, Swart and Storbeck, 2015). Metabolism proceeds via the conversion of 11OHA4 to 11KA4 by HSD11B2. 11KA4 is then preferentially converted to 11KT by AKR1C3 and 11KT can then be 5α-reduced to yield 11KDHT (Pretorius et al., 2017) (Fig. 6). Interestingly, the rate at which 11KT and 11KDHT are inactivated by prostate cancer cell lines has been shown to be significantly lower than for T and DHT, suggesting that these metabolites may remain active for longer than the classical androgens (Pretorius et al., 2016). Significantly, both 11KT and 11KDHT have been shown to be able to induce androgen-regulated gene and protein expression as well as cell growth in androgen dependent prostate cancer cell lines suggesting that these androgens may play a previously overlooked, but important role in the development and progression of CRPC (Pretorius et al., 2016). Indeed, a recent study showed that the levels of these 11-oxygenated androgens were higher than those of the classical androgens in tissue from two patients with prostate cancer (du Toit et al., 2017). Despite the promising results obtained in these initial studies, much work is still required to elucidate the contribution of these androgens to CRPC.

### 8.3 Placenta

The placenta expresses *CYP11A1* and catalyses StAR-independent (Sugawara et al., 1995) *de-novo* steroidogenesis probably facilitated by MLN64 with StAR-like activity (Bose et al., 2000). *De-novo* steroidogenesis in the placenta yields mainly PREG and PROG due to low levels of CYP17A1. However, CYP17A1 activity is sufficient for the production of T, E1 and E2, as has been demonstrated for trophoblasts (Escobar et al., 2011). In addition, the placenta possesses the enzymatic machinery capable of converting C_19_ steroids derived from the foetal adrenal and liver (DHEAS and 16αOH-DHEA, respectively) to oestrogens during their transplacental passage (Miller and Auchus, 2011). This mechanism protects the mother (and female foetus) from virilisation by the high concentrations of C_19_ steroid released into circulation by the foetal adrenal (Conte et al., 1994). However, 5α-reduced androgens cannot be aromatised and therefore can transfer from fetus to mother unhindered by the placenta, which is observed e.g. in aromatase deficiency and PORD. C_19_ steroid conversion in the placenta thus has a protective systemic function rather than an intracrine function.

## 9 Intracrine androgen metabolism in other tissues

### 9.1 Skin

Several cellular components of the interfollicular epidermis and pilosebaceous unit have the capability of *de-novo* steroid biosynthesis supported by StAR activity (Anuka et al., 2013). The resulting steroids include glucocorticoids and androgens and these pathways were reviewed in (Labrie et al., 2000, Nikolakis et al., 2016, Slominski et al., 2013). C_19_ steroids are produced from cutaneous cholesterol via the classical Δ^5^ pathway. Circulating DHEA can also be used as a precursor to produce active androgens by 3βHSD, 5α-reductase and reductive 17βHSD activity (Nikolakis et al., 2016, Slominski et al., 2013). In fact, studies with cultured sebocytes suggest that circulating DHEA may be the more important source of androgen precursors (Chen et al., 2010). Local androgen availability in the skin is necessary to stimulate sebum secretion and hair growth. The generation of DHT is essential for beard growth (Diamond et al., 1996, Messenger, 1993). Functional comparison of different epidermal cell types hint at differential functions for androgen biosynthesis and inactivation (Fritsch et al., 2001).

The presence of SRD5A1 allows for the local production of DHT (Luu-The et al., 1994), with the 5α-dione pathway bypassing T being the preferred route (Samson et al., 2010, Sugimoto et al., 1995) (Fig. 6). Acne vulgaris and androgenic alopecia are associated with local androgen hyperproduction. Acne-prone skin expresses higher levels of androgen generating enzymes than non-acne-prone skin (HSD17B3 and AKR1C3 converting A4 to T, STS and SRD5A1; summarized in (Nikolakis et al., 2016)). Local over-production of DHT has been proposed as cause of androgenic alopecia (Lee et al., 2015, Sawaya and Price, 1997) and 5α-reductase inhibitors are an established treatment for male pattern alopecia (Kaufman and Dawber, 1999).

In the skin, A4 and T can be aromatised to oestrogens, which induce beneficial effect on keratinocyte proliferation, production of extracellular matrix components and wound healing (Kanda and Watanabe, 2004a, Kanda and Watanabe, 2004b, Raine-Fenning et al., 2003). Down-stream metabolites of DHT are AST and 3α-adiol and the back conversion of the 3α-reduced metabolites is also possible. UGTs and SULTs are expressed and may contribute to phase 2 metabolism (Slominski et al., 2013). In addition, DHEA can be 7α-hydroxylated by CYP7B1, which is expressed in the skin (Hennebert et al., 2007), thereby preventing its metabolism to active androgens.

### 9.2 Salivary gland

Steroid measurements in saliva can be superior to that of serum as they rely on a non-invasive, cheap collection technique and are representative of the bioavailable, non-protein bound, levels of the steroid that can passively diffuse from circulation into saliva in the salivary glands (Vining et al., 1983). A range of liquid chromatography tandem mass spectrometry assays for salivary cortisol/cortisone and salivary androgens have thus been developed and implemented in routine clinical laboratories (Keevil, 2016). It is therefore essential that steroid metabolism in the salivary glands, which often lead to altered concentrations in saliva compared to serum concentrations, are understood in detail to correctly interpret salivary steroid concentrations in their systemic context. Salivary glands express *STS*, *SULT2B1*, *HSD3B1*, *AKR1C3*, *SRD5A1* and *CYP19A1* and the activity of the encoded enzymes allows for the conversion of circulating DHEAS and DHEA to DHT and oestrogens (Spaan et al., 2009). Low local *HSD3B1* expression coupled to derangement of subcellular compartmentalization have been suggested to lead to impaired 5α-reductase activity in Sjoergen's syndrome resulting in local DHT deficiency and oestrogen excess, as reflected by salivary concentrations (Konttinen et al., 2015, Porola et al., 2008, Spaan et al., 2009). However, *in-vitro* studies with homogenised submandibular and parotid glands showed a preference for oxidative 17βHSD activity (T → A4 etc.) (Blom et al., 1993, Djoseland et al., 1982). Oxidative HSD11B2 is present in the parotid gland (Hirasawa et al., 1997, Smith et al., 1996) resulting in a significantly higher cortisone/cortisol ratio in saliva than in serum (Hawley and Keevil, 2016) and might thus also affect the ratio of 11-keto to 11β-hydroxy androgens.

## 10 Kidney androgen metabolism and renal excretion of androgens

### 10.1 The kidney as an androgen-metabolising organ

Surprisingly the kidney has been shown to be able to catalyse the biosynthesis of active androgens using PREG as precursor for DHEA production by CYP17A1 (Quinkler et al., 2003). DHEA could be further metabolised to T, via A4 or 5α-dione, and the resulting T could be 5α-reduced to DHT. Additionally, the expression of genes (*AKR1D1* and *AKR1C2*) encoding enzymes contributing to phase 1 metabolism and inactivation of androgens have been demonstrated (Quinkler et al., 2003). It should, however, be noted that the tissue used in this study was derived from tumour nephrectomies, thus bringing into question the relevance of these results to the healthy kidney. The kidney may be the major site of the generation of the T 17α-epimer “epitestosterone” (EpiT, 17α-hydroxytestosterone), which has weak antiandrogenic functions and may inhibit certain steroidogenic enzymes. EpiT circulates with low nM concentrations and EpiT/T ratios are between 0.1 for women and up to 1 in men as measured in serum of healthy subjects by immunoassay (Havlikova et al., 2002). The ratio of urinary T and EpiT-glucuronide is used as an indicator of exogenous T administration in doping analysis (Mareck et al., 2010, Shackleton et al., 1997a, Shackleton et al., 1997b, Wada, 2015). Given its high concentrations, the physiological effects of EpiT requires further research. Furthermore, it is possible that current LC-MS/MS assays used for the determination of serum T might erroneously include EpiT in their T measurements due to limited resolution. While there is no interconversion of T and EpiT excluding T as the direct precursor to EpiT, a reductive 17αHSD (product of *AKR1C21*) has been isolated from the mouse and has been shown to convert 17-keto steroids to their 17α-hydroxyl, thus suggesting that A4 could be the direct precursor to EpiT. This enzyme was specifically expressed in the mouse kidney (Bellemare et al., 2005). Excreted metabolites of EpiT are 5α- and 5β-androstane-3α,17α-diols (Piper et al., 2009, Shackleton et al., 1997b, Wilson and Lipsett, 1966). Elimination of EpiT thus follows the same pathways as T.

The kidney has high levels of HSD11B2 activity which protects the mineralocorticoid receptor from activation by cortisol by inactivating it to cortisone (Edwards and Stewart, 1991, Funder et al., 1988). The kidney is therefore also likely a site of the conversion of 11-hydroxy C_19_ steroids to their corresponding 11-keto forms. Significantly, it is the 11-keto C_19_ steroids that are more androgenic than their 11-hydroxy precursors (Storbeck et al., 2013) and therefore while the kidney inactivates glucocorticoids it may play a vital role in activating 11-oxygenated androgens. Indeed, a recent study showed that while 11OHA4 and 11OHT are of adrenal origin, differences in the concentration of the individual 11-oxygenated steroids in the adrenal vein and inferior vena cava suggest that 11KA4 and 11KT may instead be formed in peripheral tissues (Turcu et al., 2016). The significant levels of 11KA4 measured in the serum of healthy premenopausal woman (O'Reilly et al., 2016) supports the involvement of peripheral tissue such as the kidney, especially considering that the adrenal expresses only low levels of *HSD11B2* (Coulter et al., 1999).

### 10.2 Renal excretion and analytical considerations

The main route of androgen excretion is the renal elimination of the conjugated metabolites. Sulfo- and glucoconjugates are excreted in different ratios for every androgen, while the excretion of free forms is negligible (Buiarelli et al., 2004). Renal excretion of conjugates takes advantage of (1) the hydrophilic water-soluble nature of steroid conjugates and (2) the fact that their transport across the cell membrane requires active transport mechanisms which allow for the concentration of the molecules on one side of the membrane (Pritchard and Miller, 1996, VanWert et al., 2010). Organic anion transporters are highly expressed in renal epithelia (Emami Riedmaier et al., 2012). Steroid sulfates are generally excreted at a lower rate than their glucuronidated counterparts as STS catalyse their desulfation. Besides the classical conjugates, T metabolites conjugated with cysteine have been detected in urine and plasma (Fabregat et al., 2013).

As urine collection is non-invasive it is a preferable matrix for steroid analysis. In general, total levels of metabolites (free + conjugated) are measured after de-conjugation by β-glucuronidase treatment. AST and ETIO are measured as representatives of active androgens, while DHEA and 16αOH-DHEA are measured as representatives of direct androgen precursors (Arlt et al., 2011, Krone et al., 2010). Amounts of excreted androgen and precursor metabolites from typical urinary steroid profiles excreted in a 24 h-period are summarised in Table 3 (Arlt et al., 2011, Kotlowska et al., 2017, Remer et al., 2005). Urinary 11-oxygenated AST and 11-oxygenated ETIO originate from androgen and glucocorticoid metabolism, however in different ratios. While urinary 11-oxygenated ETIO is predominantly a product of glucocorticoid metabolism, urinary 11-oxygenated AST results mainly from 11-oxygenated androgens (Jones et al., 2016, Shackleton et al., 2008). As previously mentioned, 5β-reduction (→ ETIO) is indicative of hepatic metabolism (Chen and Penning, 2014), while 5α-reduction (→ AST) takes place in most peripheral tissues (Russell and Wilson, 1994). In the context of intracrinology, 5α-reduced metabolites are thus of particular interest. The urinary steroid metabolome represents the total of all steroid biosynthetic and metabolising pathways and has proven to be a specific and sensitive biomarker for the diagnosis of steroidogenic disease (Arlt et al., 2011). De-conjugation by β-glucuronidase is time-consuming and may lead to imprecision due to variability in hydrolysis efficiency (Trabert et al., 2016), but to date only a limited number of methods for the simultaneous measurement of free, sulfated and glucuronidated androgens have been established, although significant progress has been made (Badoud et al., 2011, Doue et al., 2015, Galuska et al., 2013). Such approaches also allow the investigation of dysfunction of conjugation pathways in a single run.

**Table 3:** **Urinary concentration of C**_**19**_**steroid metabolites determined by gas chromatography mass spectrometry after de-conjugation**. All values are shown in μg/24 h. The details of the original studies are given in the footnotes below the table.

Precursor metabolites	Women	Men
DHEA	111 (57–222)^a^ 204 (82–378)^c^	396 (179–662)^a^ 355 (151–880)^c^
16αOH-DHEA	278 (188–666)^a^	
7βOH-DHEA	90 (70–100)^b^	
5-androstenediol (5-diol)	78 (47–158)^c^	151 (94–234)^c^
**Androgen metabolite**		
Androsterone (AST)	936 (733–1442)^a^ 1047^c^	2072 (1600–3067)^a^ 2138^c^
	790 (760–830)^b^	
Etiocholanolone (ETIO)	1321 (837–2041)^a^ 955^c^	2066 (1539–2468) 1549^c^
	920 (880–970)^b^	
11βOH-AST*	407 (315–655)^a^	1014 (681–1416)^a^
	380 (340–440)^b^	
11βOH-ETIO*	236 (110–375)^a^	281 (198–498)^a^
	190 (160–220)^b^	
11-keto-AST*	160 (120–190)^b^	
11-keto-ETIO*	319 (190–507)^a^	402 (317–644)^a^

*metabolite of both glucocorticoids and 11-oxygenated androgens to different extents (Shackleton et al., 2008).

a Arlt et al. (2011), median (interquartile range), 62 women and 26 men, age 18-60.

b Kotlowska et al. (2017), median (interquartile range), combined values for 25 women and 12 men, age >40.

c Remer et al. (2005), mean (interquartile range), 25 girls and 25 boys, age 17–18.

As intracrine androgen activation takes place in the cell and may start with the uptake of an androgen precursor and end with the efflux of an inactive androgen metabolite, circulating levels of active androgens are not a valid reflection of total body androgen action (O'Reilly et al., 2014b). The assessment of circulating precursors in particular A4 (Keevil, 2014, O'Reilly et al., 2014b, Pasquali et al., 2016) including 11-oxygenated C_19_ steroids (O'Reilly et al., 2016) or androgen metabolites AST-glucuronide, 3α-adiol-3-glucuronide and 3α-adiol-17-glucuronide (Labrie et al., 1997a, Labrie et al., 1997b, Vandenput et al., 2007) are thus recommended to assess androgen burden.

## 11 Conclusions

Besides its essential function for normal male sexual development (Auchus and Miller, 2012, Krone et al., 2007), androgen intracrinology plays a crucial role for physiology of peripheral tissues in both sexes. Its dysregulation can impair local and systemic metabolic homeostasis and promote sex hormone-dependent cancer, but precise mechanisms remain to be elucidated. Model systems must be carefully chosen and significant differences in adrenal development, C_19_ steroidogenesis and subsequent metabolism between humans and common animal models, i.e. rodent and non-human primates, must be taken into account. Because of the major contributions of intracrinology to the androgen levels a peripheral target cell is exposed to, the measurement of androgen precursors (O'Reilly et al., 2014b, Pasquali et al., 2016) and metabolites (Labrie et al., 2006, Vandenput et al., 2007) is recommended to assess local androgen burden and associated health risks. Therefore, more efforts are required to exploit the progress in steroid chromatography/mass spectrometry for comprehensive C_19_ steroid profiling and to systematically evaluate such profiles for the establishment of validated links to specific clinical conditions. The tissue-specificity of intracrine pathways in combination with the differential expression of hydroxysteroid dehydrogenases and SRD5A isoforms renders the inhibition of intracrine pathways a promising treatment option in addition to or as a replacement of androgen receptor blockade therapies in sex steroid-dependent cancer. The proof-of-concept has been established by aromatase inhibition in breast cancer and 5α-reductase inhibition in prostate hyperplasia (Chumsri et al., 2011, Lowe et al., 2003, McConnell et al., 1998). Additionally, androgen biosynthesis from C_21_ precursors can be reduced by CYP17A1 inhibition with abiraterone as successfully applied for the treatment of CRPC (Attard et al., 2008, de Bono et al., 2011, James et al., 2017). While androgen metabolism in reproductive tissues is well studied, less is known to date about androgen action in non-classical target tissues, such as the liver and skeletal muscle, which are major compartments responsible for metabolic regulation and metabolic health. Emerging evidence suggests a significant role of androgens in metabolic dysfunction and metabolic disease. Furthermore, the recent discovery of intracrine pathways for the metabolism of 11-oxygenated C_19_ steroids requires further investigation at both the systemic and intracrine levels, especially considering the additional complexity associated with the regulation of these steroids by HSD11B enzymes. Also, the reverse co-regulation of androgen and glucocorticoid action (SRD5A enzymes and HSD11B2 activate androgens, but inactivate glucocorticoids) is a striking phenomenon, which must be unravelled taking tissue-specific conditions into account, as both classes of hormones play key roles in metabolic regulation.

While intracrinology has come a long way since its discovery, much work is still required to understand the cell- and tissue-specific intricacies of its physiological function in both health and disease.
